# The development of novel LTA_4_H modulators to selectively target LTB_4_ generation

**DOI:** 10.1038/srep44449

**Published:** 2017-03-17

**Authors:** Caroline M. Low, Samia Akthar, Dhiren F. Patel, Stephan Löser, Chi-Tung Wong, Patricia L. Jackson, J. Edwin Blalock, Stephen A. Hare, Clare M. Lloyd, Robert J. Snelgrove

**Affiliations:** 1 Computational Drug Design Consultant, Dulwich, London SE21 8LS, United Kingdom; 2 Inflammation Repair and Development, National Heart and Lung Institute, Imperial College London, London SW7 2AZ, United Kingdom; 3 Department of Life Sciences, Imperial College London, London SW7 2AZ, United Kingdom; 4 Division of Pulmonary, Allergy and Critical Care Medicine, Department of Medicine, University of Alabama at Birmingham, Birmingham, AL 35294, USA; 5 Birmingham V.A. Medical Center, Birmingham, AL 35294, USA

## Abstract

The pro-inflammatory mediator leukotriene B_4_ (LTB_4_) is implicated in the pathologies of an array of diseases and thus represents an attractive therapeutic target. The enzyme leukotriene A_4_ hydrolase (LTA_4_H) catalyses the distal step in LTB_4_ synthesis and hence inhibitors of this enzyme have been actively pursued. Despite potent LTA_4_H inhibitors entering clinical trials all have failed to show efficacy. We recently identified a secondary anti-inflammatory role for LTA_4_H in degrading the neutrophil chemoattractant Pro-Gly-Pro (PGP) and rationalized that the failure of conventional LTA_4_H inhibitors may be that they inadvertently prevented PGP degradation. We demonstrate that these inhibitors do indeed fail to discriminate between the dual activities of LTA_4_H, and enable PGP accumulation in mice. Accordingly, we have developed novel compounds that potently inhibit LTB_4_ generation whilst leaving PGP degradation unperturbed. These novel compounds could represent a safer and superior class of LTA_4_H inhibitors for translation into the clinic.

Leukotriene A_4_ hydrolase (LTA_4_H) is an enzyme that classically functions as an epoxide hydrolase to generate leukotriene B_4_ (LTB_4_) from leukotriene A_4_ (LTA_4_)^1^,^2^. This activity operates within an intracellular compartment and is predominantly a function of leukocytes. LTB_4_ is an extremely pro-inflammatory lipid mediator that can exert its activity by binding to receptors BLT1 or BLT2^3^. LTB_4_ can drive the recruitment and activation of an array of cells including neutrophils and is thus implicated in protection against invading micro-organisms but also in the pathology of an array of diseases^4^,^5^,^6^,^7^,^8^.

Recently, we identified a secondary anti-inflammatory activity for LTA_4_H whereby it functions as an aminopeptidases to degrade the tripeptide Pro-Gly-Pro (PGP)^9^. PGP is a neutrophil chemoattractant derived from extracellular matrix (ECM) collagen via the sequential enzymatic activity of matrix metalloproteinases and prolylendopeptidase^10^. PGP functions as a neutrophil chemoattractant by mimicking key sequences found in glutamic acid, leucine, arginine^+^ (ELR^+^) chemokines and binding to CXCR1/2^11^. Since neutrophils are themselves a prominent source of the enzymes that generate PGP, it is thought that this pathway can drive a self-sustaining vicious circle of inflammation if left unchecked^12^. We have demonstrated that PGP is readily degraded during episodes of acute pulmonary inflammation by extracellular LTA_4_H to facilitate the resolution of neutrophilic inflammation, and failure of this system culminated in augmented and prolonged inflammation with exacerbated pathology and illness^9^,^13^.

Significant quantities of PGP are found in patients with chronic neutrophilic lung diseases such as chronic obstructive pulmonary disease (COPD), Cystic Fibrosis (CF) and bronchiolitis obliterans syndrome (BOS), peaking with exacerbation of disease and inversely correlating with lung function^10^,^11^,^14^,^15^,^16^,^17^. Accordingly, it seems that the LTA_4_H-PGP degradation pathway is perturbed in these chronic diseases to enable PGP to accumulate and drive inflammation^9^,^15^,^18^. Together, these studies highlight the critical importance of the secondary PGP-degrading activity of LTA_4_H.

LTA_4_H therefore represents a highly unusual enzyme with dichotomous and directly opposing pro- and anti-inflammatory activities that dictate the amplitude and persistence of neutrophilic inflammation^19^. The enzyme itself is folded into 3 domains, which manifest as N-terminal, catalytic and C-terminal domains^20^. The interface of these domains forms an L-shaped cavity where the active site of the enzyme is located. The opening part of this cavity, near the protein surface, is wider and highly hydrophilic before narrowing at the site of the catalytic zinc into a predominantly hydrophobic tunnel that penetrates deeper into the protein. The wider hydrophilic part of the cavity is the site of peptide binding, whereas LTA_4_ occupies the entire cavity with its epoxide coordinating with the zinc and its long hydrophobic tail extending down into the apolar tunnel. It is clear therefore that the opposing activities of LTA_4_H reside within distinct yet overlapping active sites, with specific amino acid residues required for each^21^,^22^.

There has been significant interest from pharmaceutical companies to target LTA_4_H therapeutically to alleviate LTB_4_-mediated pathologies, but despite the generation of several excellent inhibitors, these drugs have failed to demonstrate clinical efficacy or have been withdrawn from trials owing to deleterious side effects^23^,^24^. It is feasible that the lack of success of these compounds may be due to their failure to distinguish between the opposing roles of LTA_4_H and thus inadvertently prevent PGP degradation. Searle/Pharmacia developed the potent, orally active inhibitor SC567461A that entered clinical trials for inflammatory bowel disease, but was withdrawn owing to adverse outcomes^25^,^26^,^27^. DeCODE pharmaceuticals subsequently utilized a fragment based drug discovery program to identify inhibitors of LTA_4_H, leading to the development of the potent, orally active compound DG-051 that entered phase IIa clinical trials for myocardial infarction and stroke before further development being precluded^28^,^29^,^30^. More recently, Johnson & Johnson developed potent, orally active benzothiazole derivatives as LTA_4_H inhibitors^31^,^32^,^33^ leading to the assessment of JNJ-40929837 in a bronchial allergen challenge model of asthma^24^. Despite JNJ-40929837 demonstrating clear target engagement and reducing LTB_4_, this drug again failed to show any clinical benefit over placebo^24^.

Some studies have previously proposed that it is feasible to develop compounds that can selectively distinguish between the dual activities of LTA_4_H. Lai and colleagues suggested that diphenyl ether derivatives were capable of augmenting aminopeptidase activity against a synthetic Ala-*p*-nitroanilide substrate^34^. Accordingly, Paige and Shim have demonstrated that 4-methoxy diphenylmethane (4-MDM) augmented the aminopeptidase activity of LTA_4_H and conferred therapeutic benefit in murine models of emphysema^35^,^36^. Conversely, Haeggstrom and colleagues have suggested that it is feasible to selectively inhibit the epoxide hydrolase activity whilst leaving the aminopeptidase activity unperturbed, demonstrating that the molecule 4-(4-benzylphenyl) thiazol-2 amine (ARM1) could inhibit LTB_4_ generation without affecting PGP degradation^37^.

In this study, we have utilized robust, physiologically relevant assays to provide a definitive side-by-side comparison of existing classes of LTA_4_H modulators and develop novel selective compounds. We demonstrate that existing LTA_4_H inhibitors that have entered the clinic are equipotent at inhibiting both LTB_4_ generation and PGP degradation and *in vivo* administration can consequently result in PGP accumulation. We highlight the necessity of using the physiological substrate, PGP, when evaluating the aminopeptidase activity of LTA_4_H; with diphenyl ether derivatives failing to boost PGP cleavage as has been reported with an alternative Ala-*p*-nitroanilide substrate. Finally, we have developed two distinct chemical series of compounds that can selectively inhibit LTB_4_ generation with a selectivity that is superior to that of ARM1. These novel compounds could form the basis for the development of selective and safer therapeutics for targeting LTA_4_H in a broad array of disease modalities.

## Results

### A rational approach to selective LTA_4_H inhibitor design

**Figure 1:**
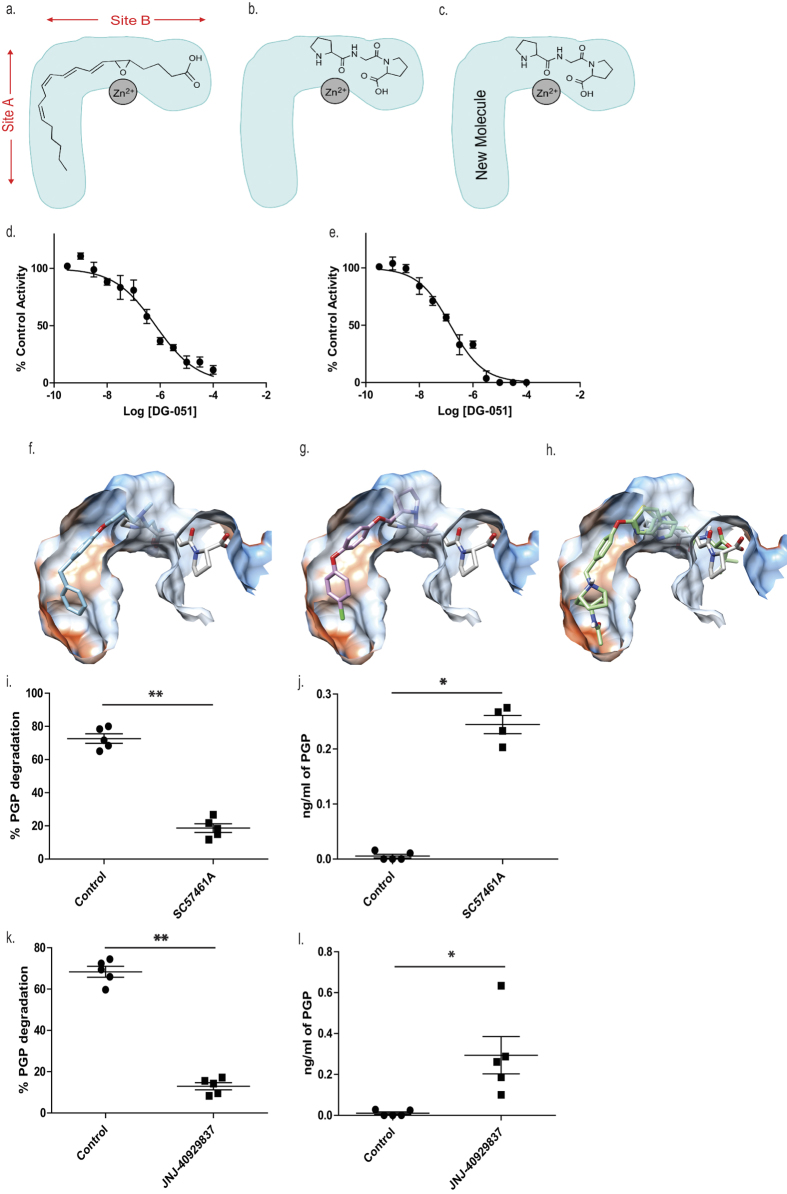
LTA_4_H inhibitors to have entered the clinic are non-selective and inhibit PGP degradation *in vitro* and *in vivo*. Schematic representation of the binding site of LTA_4_H showing the relative positions occupied by LTA_4_ (**a**) and PGP (**b**). (**c**) Proposed solution for a new compound that sits in the narrow arm occupied by the hydrophobic tail of LTA_4_, (Site A) blocking its hydrolysis, but retaining ability to cleave the tripeptide PGP (Site B). The capacity of DG-051 to inhibit LTA_4_H epoxide hydrolase (LTB_4_ generation; (**d**)) and aminopeptidase activities (PGP degradation; (**e**)). Overlay of the X-ray structures of ligand-LTA_4_H structures of (**f**) SC57461 (blue; 3U9W.pdb) and (**g**) DG-051 (pink; 3FH7.pdb) with that of the stable PGP analogue OBP-Pro (white, 4MS6.pdb). The protein surface is coloured by amino acid hydrophobicity from most hydrophobic (orange) to hydrophilic (blue) through white regions. (**h**) JNJ-40929837 (green) was docked into the protein with Autodock Vina. Mice were orally administered SC57461A (**i** and **j**) or JNJ-40929837 (**k** and **l**) and serum aminopeptidase activity (**i** and **k**) or absolute PGP concentrations (j and l) were determined. IC_50_ curves were generated from 3 technical replicates at each concentration of test compound and at least 2 biological replicates. Data for *in vivo* studies (**i**–**l**) are representative of 2 experiments with 5 mice per group. Results depicted as mean ± SEM. *P < 0.05, **P < 0.01 using Mann–Whitney statistical test.

Previous studies that have aimed to develop potent LTA_4_H inhibitors have largely failed to consider the physiological significance of the recently identified anti-inflammatory aminopeptidase activity. It is apparent that the two activities of LTA_4_H occur via distinct but overlapping active sites, both located in a deep L-shaped cleft. One arm of this cleft is narrow and hydrophobic (Site A; Fig. 1a) and the other contains the zinc-binding site (Site B; Fig. 1a). LTA_4_ fills the entire cavity, with its hydrophobic tail buried in one arm and the zinc chelated to the epoxide in the other arm (Fig. 1a). In contrast, aminopeptidase PGP-degrading activity appears confined to the zinc-containing arm (Fig. 1b). We hypothesized that whilst conventional LTA_4_H inhibitors would occupy both sites it would be preferable to develop molecules to bind to one of the two overlapping binding sites (Site A) present in LTA_4_H, leaving Site B free to bind PGP (Fig. 1c). This should block detrimental hydrolase activity, whilst preserving beneficial peptidase function.

### Conventional LTA_4_H inhibitors that have entered the clinic are non-selective and inhibit PGP degradation

To validate our hypothesis that conventional LTA_4_H inhibitors fail to distinguish between the dual activities of the enzyme, we synthesized SC57461A, DG-051 and JNJ-40929837 and assessed their capacity to inhibit LTB_4_ generation and PGP degradation. In keeping with previous literature, all were potent inhibitors of LTA_4_H epoxide hydrolase activity in a murine neutrophil-based assay (Table 1; Fig. 1d for representative IC_50_ curve for DG-051). However, each of these compounds was also comparably potent at inhibiting the aminopeptidase activity of recombinant LTA_4_H against the PGP substrate (Table 1; Fig. 1e for representative IC_50_ curve for DG-051). This lack of selectivity can be rationalized by examining published X-ray data of LTA_4_H bound to SC57461A (3U9W.pdb) and DG-051 (3FH7.pdb) with that of 1-{4-oxo-4-[(2S)-pyrrolidin-2-yl]butanoyl}- L-proline (OBP-Pro), a stable PGP analogue (4MS6.pdb). In all cases the structure of the protein is highly conserved (rms 0.28–0.35 Å over 611 atoms) and focussing on the active site of the enzyme shows that both SC57461A (Fig. 1f) and DG-051 (Fig. 1g) encroach into the zinc binding arm (Site B) that OBP-Pro, and by analogy PGP, occupies. Docking JNJ-40929837 into the same site (Fig. 1h) demonstrates that this compound fills both arms, again suggesting it will impede PGP binding. It has previously been demonstrated that oral administration of SC57461A or a JNJ-40929837 derivative to naïve mice resulted in a potent inhibition of LTB_4_ generation in an *ex vivo* whole blood assay^25^,^31^. Importantly, we demonstrate that a comparable protocol, whereby SC57461A or JNJ-40929837 are orally administered to naïve mice, also potently abrogates serum aminopeptidase PGP-degrading activity (Fig. 1i and k, respectively) and leads to serum accumulation of PGP (Fig. 1j and l, respectively). These studies highlight the potential inherent risk of these potent, yet non-selective, LTA_4_H inhibitors.

### Diphenyl ether derivatives fail to augment LTA_4_H aminopeptidase activity when utilizing the physiological substrate PGP

Previous reports have cited the potential of simple diphenyl ether derivatives to enhance aminopeptidase activity in assays using chromogenic synthetic peptide substrates^34^. The compound 4-MDM exemplifies this class of compounds, being demonstrated to augment aminopeptidase activity versus an Ala-*p*-nitroanilide substrate with an AC_50_ of 50 μM^35^. In our hands, whilst 4-MDM did promote aminopeptidase activity versus an Ala-*p*-nitroanilide substrate, this augmentation was not observed when utilizing the physiological substrate PGP; with modest inhibition even apparent (Table 1). The size and structural differences between PGP and Ala-p-nitroanilide substrates may result in their altered occupancy and positioning within the active site of LTA_4_H, and rationalize the differences observed in the behaviour of 4-MDM. However, these studies clearly demonstrate the importance of utilizing a physiological substrate rather than one that facilitates high throughput screening and suggest that diphenyl ether derivatives do not offer a promising chemical starting point as a generation of selective LTA_4_H modulators.

### Development of selective LTA_4_H inhibitors to specifically target LTB_4_ generation

Haeggstrom and colleagues recently identified ARM1 as a potential selective LTA_4_H inhibitor, with the capacity to inhibit LTB_4_ generation without perturbing PGP hydrolysis. They demonstrated that ARM1 efficiently inhibited LTB_4_ production by neutrophils with an IC_50_ of 0.5 μM without significantly compromising PGP degradation^37^. In our cell based system, ARM1 exhibited a comparable capacity to inhibit LTB_4_ generation (Table 1). However, whilst we did confirm a distinct selectivity of ARM1 in inhibiting the epoxide hydrolase activity over the aminopeptidase activity, this compound was, in our hands, capable of inhibiting PGP degradation with an IC_50_ of around 9.1 μM (Table 1). In publishing the X-ray crystallography structure of ARM1 within the active site of LTA_4_H, the authors report two potential orientations of the compounds located within Site A portraying some fluidity in binding. Our docking analysis of ARM1 within the active site of LTA_4_H revealed one of six poses where the compound sits in the peptide binding site (Supplementary Figure S1) – which may rationalize the partial inhibition of this activity.

### Resveratrol-based compounds to selectively target LTA_4_H

**Figure 2:**
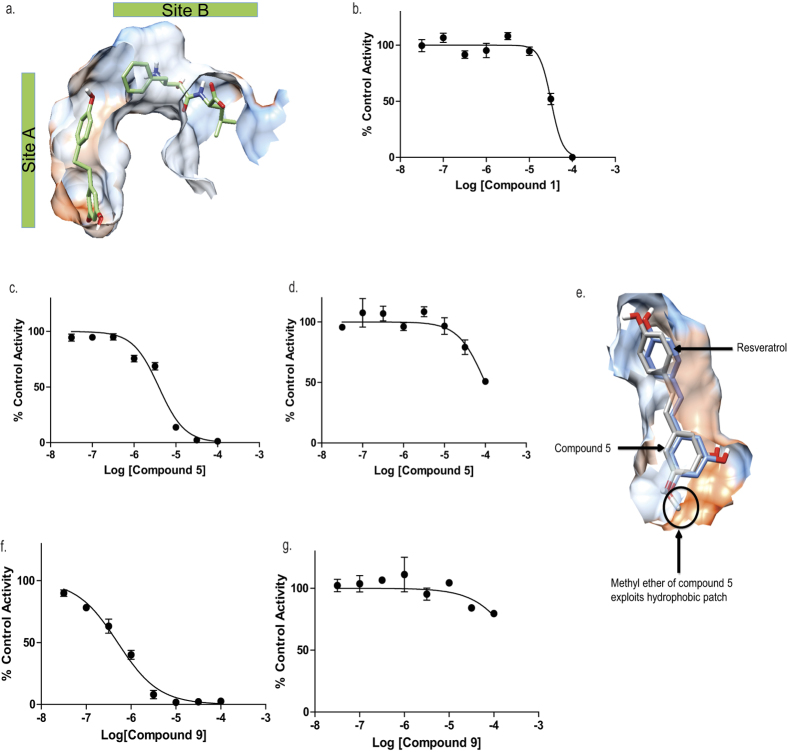
Compounds with a resveratrol core are selective epoxide hydrolase inhibitors. (**a**) LTA_4_H contains two overlapping binding Sites A and B with previous X-ray crystallography studies revealing that two small molecules are capable of binding in each of the sites simultaneously (3FTX.pdb). Peptidase inhibitor Bestatin occupies Site B and *2H*-Resveratrol sits in Site A. The protein surface is coloured by amino acid hydrophobicity from most hydrophobic (orange) to hydrophilic (blue) through white regions. LTA_4_H epoxide hydrolase activity (**b**,**c** and **f**) and aminopeptidase activity (**d** and **g**) are presented following treatment with resveratrol (compound 1; **b**), compound 5 (**c** and **d**) and compound 9 (**f** and **g**). (**e**) Site A with docked pose of pinostilbene (compound 5; grey) overlaid on the X-ray structure of *2H*-resveratrol bound to LTA_4_H (blue; 3FTS.pdb). The protein surface is coloured by amino acid hydrophobicity from most hydrophobic (orange) to hydrophilic (blue) through white regions. The methyl ether exploits a hydrophobic patch in Site A, an interaction that is not favoured for Resveratrol and accounts for the increase in activity of compound 5 compared to Resveratrol. IC_50_ curves were generated from 3 technical replicates at each concentration of test compound and at least 2 biological replicates. Results depicted as mean ± SEM.

A published X-ray structure of LTA_4_H (3FTX.pdb) shows two different molecules simultaneously bound in each arm of the active site (Fig. 2a). A molecule of *2H*-resveratrol solely occupies our target site (Site A), whilst one of bestatin – a known peptidase inhibitor, occupies the Zn catalytic site (Site B). The molecule resveratrol (**compound 1**) occupies the same region of the active site (Site A) as *2H*-resveratrol, but with its double bond reducing flexibility (3FTS.pdb). In this structure, the R2 phenol hydroxyl of resveratrol makes a key interaction with a hydrophilic patch on the enzyme, encompassing Ser-379, Lys-364 and Asp-312. Thus resveratrol (**compound 1**) seemingly represented a rational starting point for the development of a novel selective LTA_4_H inhibitor. In accordance with the binding studies, resveratrol (**compound 1**) exhibited minimal inhibition of PGP degradation, with negligible inhibition observed at a concentration of 100 μM (Table 2a). Furthermore, this compound exhibited a modest inhibition of LTB_4_ generation and thus displays some potential to distinguish between the two functions of the enzyme with biased inhibition of the epoxide hydrolase activity (Fig. 2b and Table 2a). An iterative approach of targeted drug design and structure activity relationship studies (SAR) was subsequently utilized to develop compounds based upon the resveratrol core that would exhibit superior inhibition of LTA_4_H epoxide hydrolase activity whilst maintaining selectivity.

Changing the configuration of the double bond from *trans*- (resveratrol; **compound 1**) to *cis*- (**compound 2**) alters both the distance and relative dispositions of the two aromatic rings but seemingly had minimal impact on the compound’s capacity to inhibit epoxide hydrolase or aminopeptidase activities of LTA_4_H (Table 2a). The results of docking experiments suggest both *cis*- (**compound 2**) and *trans*-resveratrol (**compound 1**) occupy the desired region (Site A) of the active site with one of the phenol hydroxyls (R2) of both compounds forming an identical interaction with a hydrophilic patch on the enzyme. Blocking all three hydroxyls of trans-resveratrol with methyl ethers (**compound 3**) or acetyl groups (**compound 4**) modestly reduced the capacity to inhibit LTB_4_ generation (Table 2a). However, methylation of a single hydroxyl (R1) gives a compound with significantly increased capacity to inhibit LTB_4_ generation (**compound 5**. Figure 2c and Table 2a), whilst still leaving aminopeptidase activity largely unperturbed (Fig. 2d and Table 2a). Docking this molecule into Site A shows that this can be attributed to an interaction with a hydrophobic patch that cannot be exploited by the polar R1 OH of resveratrol (Fig. 2e).

**Figure 3:**
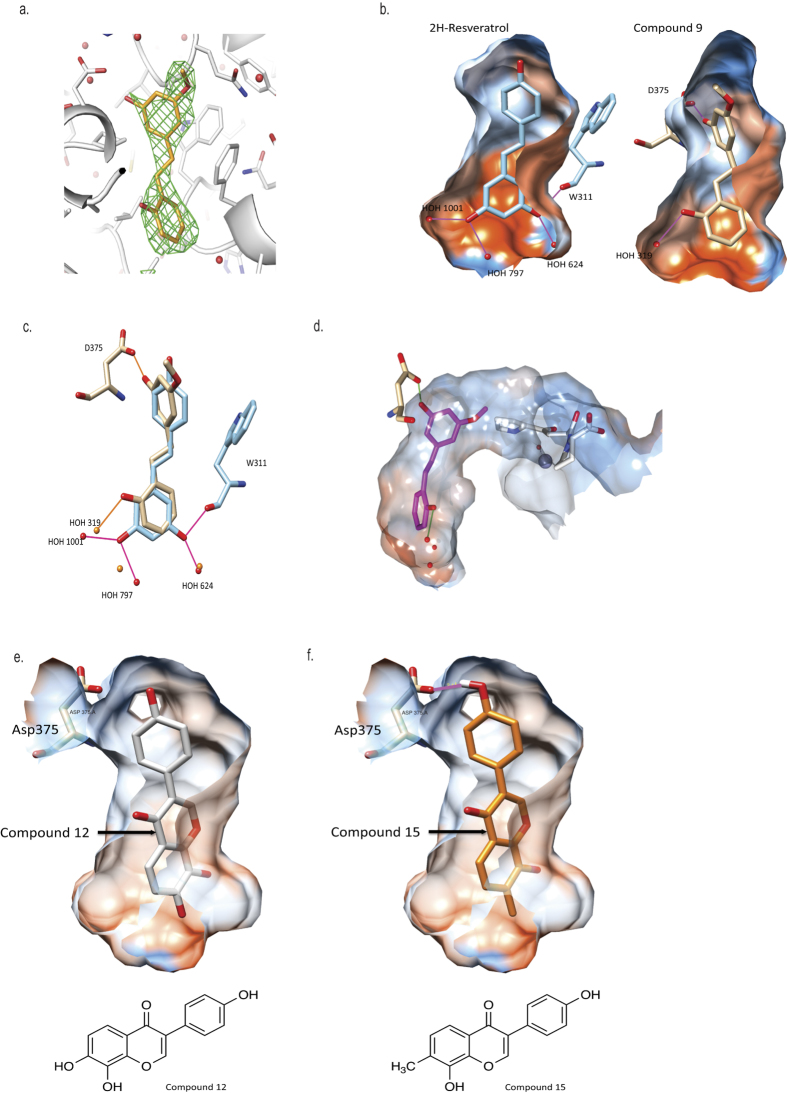
Compounds 9 and 15 make a hydrogen bond with Asp-375 within Site A of the active site of LTA_4_H. (**a**) Green mesh surrounding Compound 9 (orange) shows the Fo-Fc difference map density (2σ level) having omitted the compound from the model. (**b**) Comparison of X-ray structure of *2H*-resveratrol (blue; 3FTU.pdb) and compound 9 (bronze; our study) in Site A of LTA_4_H. The protein surface is coloured by amino acid hydrophobicity from most hydrophobic (orange) to hydrophilic (blue) through white regions. (**c**) Relative positions of *2H*-resveratrol (blue) and compound 9 (bronze) showing hydrogen bonding interactions. *2H*-resveratrol H-bonds to the carbonyl of Trp-311 and 3 water molecules (pink) and its position is further reinforced by a π-stacking interaction with the indole of Trp-311. Compound 9 adopts a head-to-tail orientation such that its hydroxyl groups form H-bonds to Asp-375, key for hydrolase activity, and a single water molecule (orange). (**d**) Compound 9 (pink) does not impinge significantly on the peptidase site delineated by OBP-Pro (white; 4MS6.pdb) when the X-ray structures of Compound 9-LTA_4_H (our study) and 4MS6.pdb are overlaid. Best scored poses of compound 12 (**e**; white) and compound 15 (**f**; orange) docked into site A of LTA_4_H with Autodock Vina. The R2 OH of compound 12 is located in a hydrophobic region so replacing this group with a methyl substituent (compound 15) improves interaction with the protein. In addition, a hydrogen-bonding interaction between the phenol of compound 15 and Asp-375 is also observed.

Further modification of **compound 5** confirmed that the double bond between the two aromatic rings was not essential for inhibition of epoxide hydrolase activity (**compound 6**; Table 2b). Moreover increasing the size of the alkyl ether substituent at R1 from methyl to ethyl (**compound 7**) did not offer any advantage in promoting LTB_4_ inhibitory capacity (Table 2b). Exploring other regioisomers of **compound 6** (Table 2b) highlighted that the positions of the phenolyic hydroxyl groups were not optimal. For example, moving the phenol hydroxyl from the R5- to the R4-position (**compound 8**) had modest impact on its capacity to inhibit LTB_4_ generation, but relocation of the hydroxyl to the R3-position (**compound 9**) augmented the LTB_4_ inhibitory capacity of the compound 10-fold (IC_50_ 0.5 μM; Fig. 2f and Table 2b), whilst still leaving the aminopeptidase PGP-degrading activity unperturbed (Fig. 2g and Table 2b). The origins of the augmented capacity of **compound 9** to inhibit LTA_4_H epoxide hydrolase activity were explored by modelling and X-ray crystallography (Fig. 3a). The X-ray structure of LTA_4_H shows high similarity to literature examples (RMSD between 606 atom pairs is 0.204 Å). Of most note, **compound 9** has flipped within Site A of the active site of LTA_4_H relative to *2H*-resveratrol (3FTU.pdb) so that the phenolic hydroxyl in the 2-position now forms a hydrogen bond with Asp-375 (Fig. 3b and c) and the two ligands are effectively located head-to-tail. This pose is also present in those generated by *in silico* docking. Whilst *2H*-resveratrol forms two interactions with Trp-311, one H-bond and one π-stack, these are absent in the **compound 9** structure, where the 3-methoxy-5-phenol ring rotates to allow the phenol to H-bond to Asp-375 (Fig. 3c). Importantly, previous site-directed mutational interrogation of the active site of the enzyme has identified Asp-375 to be the only residue uniquely and absolutely required for the enzyme’s epoxide hydrolase activity^38^. This therefore likely underlies the far superior capacity of **compound 9** versus *2H*-resveratrol to inhibit that capacity of LTA_4_H to generate LTB_4_. Overlaying our X-ray data of **compound 9** bound within the active site of LTA_4_H with the published structure of OBP-Pro (4MS6.pdb) demonstrates that **compound 9** occupies our target site A, without encroaching into the zinc binding arm (site B) that OBP-Pro, and by analogy PGP, occupies (Fig. 3d).

Thus **compound 9** represented a chemical series with clear and rational potential to selectively inhibit the epoxide hydrolase activity of LTA_4_H. We next questioned whether this compound would exhibit a comparable capacity to inhibit LTB_4_ generation in human neutrophils. Preliminary data obtained using blood-derived neutrophils from two distinct donors demonstrated that compound 9 inhibited LTB_4_ generation with an IC_50_ of 2.4 μM – comparing favourably with that obtained with murine cells. Furthermore, it should be stressed that human neutrophils produced more LTB_4_ on a per cell basis than mouse neutrophils in our assays, and thus all compounds were slightly less potent in the human system. To determine whether **compound 9** possessed suitable drug-like properties to merit advancement, *in vitro* ADMET studies were performed to assess plasma protein binding, microsome stability and logD (Supplementary Table S1). These studies highlighted that a potential weakness of **compound 9** was that it was readily metabolized in microsomes and would thus be readily cleared *in vivo*.

### Isoflavone-based compounds to selectively target LTA_4_H

At this point it became clear that we needed to improve the drug-like properties of our series. Given the size restraints imposed by the binding site, our initial investigations focussed on identifying alternative groups to link the two aromatic rings. We found that amides lost all capacity to inhibit either activity but replacing the carbon adjacent to ring A with a ketone (**compound 10**) was tolerated showing preferential inhibition of the epoxide hydrolase activity (Table 3). Moreover, the isoflavone daidzein (**compound 11**), which incorporates the structure of **compound 10**, was equipotent with its uncyclized counterpart in terms of selective inhibition of the hydrolase activity (Table 3) and represents a new chemotype, with the additional benefit of greatly increased metabolic stability reported (t_1/2_ 7.75 ± 0.36 h)^39^. A survey of commercially available isoflavones rapidly identified 7,8,4′-trihydroxyisoflavone (**compound 12**) with >8-fold selectivity for hydrolase over peptidase inhibition (Table 3). Docking **compound 12** into the active site of LTA_4_H produces poses in which the new compound occupies the same region as resveratrol and in which the R3 hydroxyl makes a key interaction with the same hydrophilic patch of the enzyme (Fig. 3e); and the parallel behaviour of the two series supports this hypothesis. In sharp contrast to **9**, we determined that the isoflavone **compound 12** showed greatly increased metabolic stability over the resveratrol series in both mouse and human microsomes (Supplementary Table 1).

Investigating the role of the hydroxyl substituents demonstrated that loss of the R1 and R3 hydroxyls from **compound 12** (to give **compound 13**) diminished the capacity of the compound to inhibit the epoxide hydrolase activity of LTA_4_H, with docking studies suggesting that the R3 phenyl OH was no longer capable of finding the key binding interaction with a hydrophilic patch of the enzyme identified in the earlier structure-activity studies. Blocking both R3 and R2 hydroxyls by formation of the acetal (**compound 14**) also produced a modest reduction in hydrolase inhibition versus **compound 12**, whilst also increasing aminopeptidase inhibition (Table 3). However, replacing just the R2 hydroxyl of **compound 12** with a methyl group (**compound 15**) gives a compound with increased ability to inhibit hydrolase activity, whilst showing clear selectivity versus aminopeptidase inhibition (Table 3). Docking studies rationalized this enhanced inhibition of epoxide hydrolase activity, with the R2 methyl making an additional interaction with a hydrophobic patch within the active site, whilst still allowing the R3 OH to hydrogen bond to the aforementioned hydrophilic region (Fig. 3f). Importantly, docking studies strongly suggest that the R1 OH of **compound 15** is now able to make a hydrogen bond with Asp-375 (Fig. 3f), further rationalizing the potency and specificity with which it is able to inhibit the epoxide hydrolase activity of LTA_4_H. Thus **compound 15** offered another chemical starting point for the development of selective LTA_4_H inhibitors but with greater metabolic stability.

## Discussion

LTA_4_H inhibitors developed by pharmaceutical companies to alleviate LTB_4_-mediated pathologies have demonstrated clear target engagement, but failed to show efficacy in the clinic. In this study, we validated that clinically evaluated LTA_4_H inhibitors target both activities of the enzyme and importantly enable PGP accumulation *in vivo*, highlighting the necessity to generate selective compounds. Furthermore, we reveal that diphenyl ether derivatives purported to augment the aminopeptidase activity of LTA_4_H are ineffective when using the physiological substrate PGP. Finally, we identify two distinct chemical series that can selectively inhibit LTB_4_ generation whilst maintaining PGP degradation and may offer a starting point for the development of novel, superior and safer LTA_4_H modulators.

SC57461A, DG-051 and JNJ-40929837 have all been reported as potent, selective, orally efficacious inhibitors of LTB_4_ synthesis. However, we report for the first time that these compounds are equipotent at inhibiting PGP degradation, and rationalized this in terms of their encroachment into the peptide binding arm of the active site. We have previously reported the absolute requirement of LTA_4_H to degrade PGP *in vivo* utilizing knock out animals^9^,^13^. However, a valid caveat has been that LTA_4_H inhibitors may not yield comparable results to gene knock outs, with sufficient residual enzyme activity remaining to degrade PGP following inhibitor administration. It is telling therefore, that oral administration of SC57461A or JNJ-40929837 to naïve mice potently inhibited aminopeptidase activity and led to serum accumulation of PGP. PGP is anticipated to support persistent neutrophilic inflammation and ensuing pathology in chronic lung diseases such as COPD, CF and BOS where it inversely correlates with lung function, spikes with exacerbations and is reduced with in-patient therapy^10^,^11^,^14^,^15^,^16^,^17^. Furthermore, chronic administration of PGP alone into the lungs of mice is sufficient to elicit an emphysematous phenotype and neutralization of PGP can ameliorate cigarette smoke-induced pulmonary inflammation in mice^11^,^40^. Additionally, we have demonstrated that failure to degrade PGP in response to a relatively innocuous pulmonary bacterial challenge results in a more pronounced neutrophilic infiltrate, protease imbalance, ECM attack and enhanced illness and pathology – all hallmarks of aforementioned chronic lung disease characterized by persistent PGP^13^. Thus it is clearly apparent that perturbation of the LTA_4_H pathway in a manner that can facilitate PGP accumulation carries significant inherent risks. Accordingly, it is clear that we should be extremely vigilant to adverse effects of LTA_4_H inhibitors and novel selective drugs that can spare PGP degradation would be desirable.

More recent studies have reported the potential to pharmacologically manipulate the individual activities of LTA_4_H^34^,^35^,^36^. Indeed, 4-MDM has been reported to ameliorate disease in murine models of emphysema triggered by intranasal administration of elastase or long term cigarette smoke exposure by purportedly augmenting LTA_4_H aminopeptidase activity^35^,^36^. It is noteworthy however, that these studies have not assessed the capacity of 4-MDM to directly augment aminopeptidase activity of LTA_4_H against the physiological substrate PGP, instead using chromogenic amino acid analogues. In our hands, 4-MDM failed to augment PGP degradation, highlighting the importance of assaying enzyme activity using physiological, albeit more laborious, methodologies as opposed to those that enable high throughput screening. The success of 4-MDM in preclinical animal models, in light of these findings, is difficult to rationalize but could suggest an alternative peptide substrate for LTA_4_H or off target effects of 4-MDM given its likely low specificity and the substantial doses administered.

Recently, the Haeggstrom group elegantly demonstrated that the molecule ARM1 occupied the end of the L-shaped hydrophobic cavity, whilst still permitting a PGP analogue (OBP-Pro) to occupy the zinc binding site^37^. Accordingly, they showed that ARM1 could inhibit LTB_4_ generation by neutrophils with an IC_50_ of ~0.5 μM, whilst exhibiting no inhibition of PGP degradation at a concentration of 100 μM. In our experimental system, ARM1 exhibited a comparable capacity to inhibit LTB_4_ generation in cell based assays but also exhibited some capacity to inhibit PGP degradation - albeit still with marked selectivity towards inhibition of hydrolase activity. The discrepancy is almost certainly attributable to differing methodologies that are applied. When assessing aminopeptidase activity of LTA_4_H versus PGP we have utilized enzyme at a concentration of 0.1 μg/ml, in keeping with physiological levels of the enzyme found within the blood, whereas the Haeggstrom group have utilized 5 μg/ml LTA_4_H. Whilst the merits of subtle differences in experimental methodologies can be argued, the direct side-by-side interrogation of compounds in identical assays, as performed in our study, enabled us to confidently assess relative potencies and selectivity of these drugs. To our mind ARM1 was the only compound tested that exhibited a marked selectivity between enzyme activities and thus represented the gold standard to which our novel drugs should be compared.

Subsequently, we have developed two distinct chemical series that exhibit the capacity to inhibit LTA_4_H-mediated LTB_4_ generation whilst demonstrating exquisite selectivity and leaving the aminopeptidase activity largely unperturbed. **Compound 9** exhibited sub-μM capacity to inhibit LTB_4_ generation in a cell based assay, whilst showing no inhibition of PGP degradation at 100 μM – thus exhibiting a superior selectivity to ARM1 in our hands. Rationalizing this selective inhibition, X-ray crystallography demonstrated that **compound 9** formed a hydrogen bond with Asp-375. Previous site-directed mutagenesis studies have demonstrated that Asp-375 was absolutely required for hydrolysis of LTA_4_ into LTB_4_ but not for the peptidase activity – the only residue reported to be capable of making this distinction^38^. Whilst **compound 9** exhibited a targeted potency and selectivity, preliminary studies suggest that it does not possess a suitable drug profile, being readily metabolized within microsomes. Thus whilst **compound 9** represents a useful biological tool, further development would be mandatory to enhance *in vivo* stability. Subsequently, isoflavones were demonstrated to exhibit remarkable stability whilst still displaying a selective capacity to inhibit LTB_4_ generation. As a lead compound **15** was capable of inhibiting LTA_4_H epoxide hydrolase activity at the low μM range with minimal inhibition of aminopeptidase activity. This could again be rationalized in part by a predicted interaction with key epoxide hydrolase residue Asp-375. Thus **compound 15** also represents an exciting candidate for further drug development. Within the isoflavone series, compound 12 contains a catechol that is recognized as a PAINS substructure. However, as this is the only example of such a feature within the isoflavone series, the capacity of this class of compounds to inhibit LTB_4_ generation is clearly specific. Nonetheless, it would be prudent to be vigilant to the presence of PAINS substructures in developing the isoflavone series towards the clinic.

Drug failures are common, multifactorial and often difficult to explain. In the case of LTA_4_H, however, the lack of clinical success could feasibly be attributable to the inadvertent inhibition of the enzyme’s anti-inflammatory aminopeptidase activity. Accordingly, the compounds identified within this study could act as starting points for the development of superior and safer LTA_4_H inhibitors to selectively target LTB_4_-mediated pathologies. However, whilst this study provides clear proof-of-concept for a rationalized selective LTA_4_H inhibitor, the compounds identified require significantly more optimization to generate inhibitors with a suitable drug profile that can be translated to the clinic. Clearly, there is a need to further assess the capacity of these compounds to inhibit LTB_4_ generation in a human cell based system, preferably with disease relevance. Furthermore, the studies detailed within this manuscript highlight the clear necessity to develop compounds with a superior ADMET/DMPK profile, and we have not as yet assessed selectivity of these compounds – pertinent given the mandatory small size of the drugs. If these issues can be overcome, however, there is a clear rationale to test these classes of selective LTA_4_H inhibitors in pre-clinical animal models and ultimately the clinic.

## Methods

### Compound procurement

**Compound 1** (Resveratrol; 3,4′,5-Trihydroxy-*trans*-stilbene; 5-[(1*E*)-2-(4-Hydroxyphenyl)ethenyl]-1,3-benzenediol), **compound 4** (Triacetyl resveratrol; (5-[(1E)-2-[4-(acetyloxy)phenyl]ethenyl]-1,3-Benzenediol-1,3-diacetate, 3,5,4′-Tri-O-acetylresveratrol), **compound 5** (Pinostilbene hydrate; 3,4′-Dihydroxy-5-methoxy-trans-stilbene hydrate, 3-[(1E)-2-(4-hydroxyphenyl)ethenyl]-5-methoxyphenol hydrate), **compound 10** (1-(2,4-Dihydroxyphenyl)-2-(4-hydroxyphenyl)ethanone) and **compound 12** (7,8,4′-trihydroxyisoflavone) were all purchased from Sigma Aldrich (Dorset, UK). **Compound 2** (cis-Resveratrol) and c**ompound 3** (trans-trismethoxy Resveratrol; (E)-5-[2-(4-hydroxyphenyl)ethenyl]-1,3-benzene diol) were purchased from Cayman Chemicals (Michigan, USA). **ARM1** (4-(4-benzylphenyl)-1,3-thiazole-2-amine), **compound 11** (7-hydroxy-3-(4-hydroxyphenyl)-4*H*-chromen-4-one) and **compound 13** (7-hydroxy-3-phenyl-4*H*-chromen-4-one) were all purchased from VitasMLab (Kowloon, Hong Kong). **SC57461A** was purchased from Tocris Bioscience (Bristol, UK) and **4**-**Methoxydiphenylmethane** (4-MDM) was purchased from Fluorochem, (Hadfield, UK). **JNJ**- **40929837** and **DG**-**051** were synthesized by Peakdale Molecular (Derbyshire, UK) as previously described^28^,^41^. All other compounds were custom synthesized by Peakdale Molecular as described in Supplementary Information. ADMET (absorption, distribution, metabolism, and excretion – toxicity) studies were also outsourced to Peakdale Molecular.

### Molecular Modelling

Structures were analysed and visualized using UCSF Chimera (1.9–1.10) and docking experiments were run using this implementation of AutoDock Vina. The 1.25 Å resolution X-ray structure of human LTA_4_H in complex with SC57461 (3U9W.pdb) was prepared by running Dock Prep to add hydrogens, charges, and correct incomplete sidechains. Ligands were built in Chimera and prepared using the inbuilt ligand preparation script. AutoDock Vina was configured to generate 9 binding modes; the “Exhaustiveness of search” parameter was unchanged from the default of 8 and the maximum energy score range allowed for poses was set to 3 kcal/mol. A box 15 × 8 × 15 Å was set up to define the receptor search volume and the results analyzed with ViewDock.

Ligand-protein structures were compared by aligning the protein chains of the reference and match structure(s) using MatchMaker as implemented in Chimera. Chimera is developed by the Resource of Biocomputing, Visualization, and Informatics at the University of California, San Francisco (supported by NIGMS P41-GM103311).

### Crystallography methods

LTA_4_H (Novoprotein, NJ, USA) at 6 mg/ml in 10 mM Tris pH 8.0, 25 mM KCl was crystallized at 18 °C by hanging drop vapour diffusion against a reservoir of 20% PEG 8000, 0.1 M Sodium Acetate, 0.15 mM Imidazole pH7.8, 5 mM YbCl_3_. Crystals were transferred to drops containing well solution supplemented with 20% (v/v) glycerol and saturating concentration (<10 mM) of **compound 9** for 3 hrs, before being flash frozen in liquid nitrogen. Data were collected on beamline I02 at Diamond Light Source (Oxfordshire, UK), and processed with Mosflm and Scala of the ccp4 suite^42^,^43^,^44^. The structure was solved using pdb 5AEN as an initial model and refined using refmac and phenix iterated with manual model building using coot^45^,^46^,^47^ to give a final structure with an R_free_ of 19.6% and good geometry (Supplementary Table S2). A stereo image showing electron density around compound 9 is depicted in Supplementary Figure S2. The coordinates and structure factors for LTA_*4*_H:Compound 9 have been deposited in the protein databank (PDB ID 5N3W).

### Mouse procedures

This study was carried out in accordance with the recommendations in the Guide for the Use of Laboratory Animals of Imperial College London. All animal procedures and care conformed strictly to the UK Home Office Guidelines under the Animals (Scientific Procedures) Act 1986, and the protocols were approved by the Home Office of Great Britain. Six- to eight-week-old female BALB/c mice were purchased from Harlan Olac Ltd (Oxon, UK). All mice were kept in specified-pathogen-free conditions and provided autoclaved food, water and bedding.

For murine *ex vivo* analysis of LTB_4_ production and PGP degradation, SC57461A (in physiological saline with 2% DMSO and 1% Tween 80), JNJ-40929837 (in 20% HPβCD) or relative vehicle controls were administered orally (10 mg/kg) to female BALB/c mice. Blood was obtained from mice 4 h after oral dosing and downstream analysis performed blinded by an independent researcher. To assess inhibition of epoxide hydrolase activity, blood (90 μl added to 10 μl 500 U/ml heparin) was diluted 1:1 in RPMI 1640 medium, after which 200 μl aliquots of the diluted blood were added to a microtiter plate. Calcium ionophore A-23187 (20 μg/ml final concentration) was added and the mixture incubated 20 min at 37 °C in a humidified incubator. The reaction was terminated by centrifugation (600 × *g*, 5 min, 4 °C). Supernatants were analyzed for LTB_4_ by EIA (see below). To assess inhibition of aminopeptidase activity, PGP degrading capacity of serum was assessed as detailed below. Furthermore, absolute concentrations of PGP in serum was determined as detailed below.

### Cell isolation

To isolate murine bone marrow neutrophils, the femur and the tibia from both hind legs were removed and freed of soft tissue attachments, and the extreme distal tip of each extremity cut off. HBSS containing 15 mM EDTA and 30 mM HEPES was forced through the bone with a syringe. After dispersing cell clumps and passage through a 100 μM sieve (BD labware, New Jersey), red blood cells were lysed by resuspending pellets in ACK buffer (for 3 minutes at room temperature) before centrifugation (800 × *g*, 5 min) and washing with HBSS. The cell suspension was centrifuged (800 × *g*, 5 min, 4 °C) and resuspended in RPMI. The cells were layered onto a 72%, 64%, 52% Percoll gradient (Sigma-Aldrich, UK) diluted in PBS (100% Percoll = nine parts Percoll and one part 10x PBS), and centrifuged (1500 × *g*, 30 min, room temperature) without braking. The neutrophils at the 64%/72% interface were harvested and washed with 20 ml RPMI. Cell viability was assessed by trypan blue exclusion and confirmed to be >90% neutrophils by flow cytometry.

To isolate human neutrophils, peripheral blood was taken from healthy volunteers following informed consent and according to an approved protocol. Neutrophils were subsequently isolated using the MACSxpress™ Neutrophil Isolation Kit (Miltenyi Biotec, UK).

### Flow cytometry

Single-cell suspensions were stained for surface markers in PBS containing 0.1% sodium azide and 1% BSA for 30 min at 4 °C and fixed with 2% paraformaldehyde. Data was acquired on a BD FACS Fortessa machine (BD Biosystems, UK). Forward scatter and side scatter gates were used to exclude debris and dead cells were excluded using a fixable near IR dead cell stain kit for 633 or 635 nm excitation. Neutrophils were defined as Ly-6G^high^ (Ly6G-FITC; BD Biosciences; clone 1A8), CD11b^high^ (CD11b-PerCP; eBioscience; clone M1/70), CD11c^low^ (CD11c-APC; BD Biosciences; clone HL3), F4/80^low^ (F4/80-PE/Dazzle; BD Biosciences; clone BM8).

### Liquid chromatography-electrospray-ionization-tandem mass spectrometry (ESI-LC/MS/MS) for PGP detection

For peptide quantification in serum, PGP and AcPGP were measured using a MDS Sciex API-4000 spectrometer (Applied Biosystems, Foster City, CA) equipped with a Shimadzu HPLC (Columbia, MD). For peptide quantification from degradation experiments, PGP and AcPGP were measured using a Thermo Accela Pump and Autosampler coupled to a Thermo TSQ Quantum Access. HPLC was done using a 2.0 × 150 mm Jupiter 4u Proteo column (Phenomenex, Torrance, CA) with A: 0.1% HCOOH and B: MeCN + 0.1% HCOOH: 0 min–0.5 min 5% buffer B/95% buffer A, then increased over 0.5–2.5 min to 100% buffer B/0% buffer A. Background was removed by flushing with 100% isopropanol/0.1% formic acid. Positive electrospray mass transitions were at 270–70, 270–116 and 270–173 for PGP and 312–140 and 312–112 of AcPGP. Peak area was measured, and PGP/AcPGP peptide concentrations were calculated using a relative standard curve method as previously described^11^.

### Assessment of LTA_4_H aminopeptidase activity

Mouse serum (diluted 1/10 in PBS) or recombinant human LTA_4_H (0.1 μg/ml; Cayman Chemicals, Michigan, USA) were incubated with 0.4 mM PGP at 37 °C in 5% CO_2_ for varying periods of time. Concentrations of PGP remaining were subsequently quantified by ESI-LC/MS/MS (as discussed above) by comparison with PGP standards. The percentage of peptide degraded was determined relative to control samples of 0.4 mM PGP alone. PGP degradation was also assessed by measurement of free proline released and its ensuing reaction with Ninhydrin (as discussed below). Test compounds were assayed in a blinded manner by an investigator during determination of the effect on LTA_4_H aminopeptidase activity.

To assess free proline liberated, aliquots from PGP degradation experiments were diluted 1 in 10 in PBS (to a final volume of 250 μl). Glacial acetic acid (250 μl) was then added, followed by 250 μl of ninhydrin solution (25 mg/ml in acetic acid/6 M phosphoric acid; heated at 70 °C to dissolve). The reaction mixture was heated at 100 °C for 60 minutes, allowed to cool to room temperature and the proline containing fraction extracted with 500 μl of toluene and optical density measured at 520 nm.

### Assessment LTA_4_H epoxide hydrolase activity

1 × 10^5^ murine bone marrow-derived neutrophils or human blood-derived neutrophils (compound 9 only) in 100 μl of RPMI were treated with A23187 at a final concentration of 20 μg/ml. 1% DMSO was used as a control. Cells were incubated for 20 minutes at 37 °C, and the reaction was terminated by centrifugation (600 × *g*, 5 min, 4 °C). Supernatants were removed and stored at −80 °C for subsequent LTB_4_ analysis. The concentration of LTB_4_ generated in the epoxide hydrolase activity assay was assayed using an EIA, according to manufacturer’s directions (R&D systems, Minneapolis, MN). Test compounds were assayed in a blinded manner by an investigator during determination of effect on LTA_4_H epoxide hydrolase activity.

### Statistical analysis

Data were analyzed using Prism 4 (GraphPad Software Inc. La Jolla, CA, USA). Data was non-parametric and statistical significance was calculated using an unpaired Mann-Whitney test (two-tailed). All p-values of ≤0.05 (*) and ≤0.01 (**) were considered significant and are referred to as such in the text.

## Additional Information

**How to cite this article**: Low, C. M. *et al*. The development of novel LTA_4_H modulators to selectively target LTB_4_ generation. *Sci. Rep.* **7**, 44449; doi: 10.1038/srep44449 (2017).

**Publisher's note:** Springer Nature remains neutral with regard to jurisdictional claims in published maps and institutional affiliations.

## Supplementary Material

Supplementary Information

## Figures and Tables

**Table 1:** Effect of LTA_4_H inhibitors to have entered the clinic and purportedly selective modulators on inhibition (IC_50_) of the epoxide hydrolase (LTB_4_ generation) and aminopeptidase (PGP degradation) activities of LTA_4_H in our assays, as described in Materials and methods.

Compound	Epoxide Hydrolase IC_50_ ± SEM (nM)	Aminopeptidase IC_50_ ± SEM (nM)
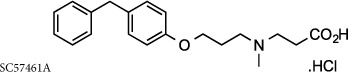	110 ± 23.5	74 ± 7.3
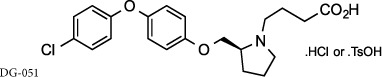	680 ± 10.0	150 ± 19.4
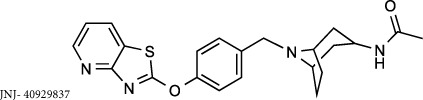	9.0 ± 0.09	9.4 ± 1.3
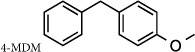	48,900 ± 9,538	75,210 ± 9,752
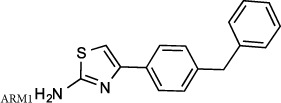	600 ± 66.2	9,110 ± 1,092

IC_50_ curves were generated from 3 technical replicates at each concentration of test compound and at least 2 biological replicates. Results depicted as mean ± SEM.

**Table 2:** Effect of (a) analogues of resveratrol (**compound 1**) and (b) (hydroxyphenyl)ethylphenols on inhibition (IC_50_) of the epoxide hydrolase (LTB_4_ generation) and aminopeptidase (PGP degradation) activities of LTA_4_H as described in Materials and methods.

Cmpd. No.	R_1_	R_2_	R_3_	Epoxide Hydrolase IC_50_ ± SEM (μM)			Aminopeptidase IC_50_ ± SEM (μM)	
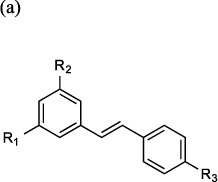								
1	OH	OH	OH	32.2 ± 5.9			>100	
2^a^	OH	OH	OH	14.3 ± 2.1			85.3 ± 33.9	
3	OMe	OMe	OMe	41.2 ± 5.5			68.8 ± 9.9	
4	OCOMe	OCOMe	OCOMe	38.6 ± 5.9			>100	
5	OMe	OH	OH	3.9 ± 0.4			99.5 ± 22.6	
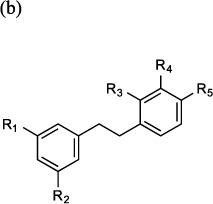								
**Cmpd**. **No**.	**R**_**1**_	**R**_**2**_	**R**_**3**_	**R**_**4**_	**R**_**5**_	**Epoxide Hydrolase IC**_**50**_** ± SEM** (**μM**)		**Aminopeptidase IC**_**50**_** ± SEM** (**μM**)
6	OMe	OH	H	H	OH	6.4 ± 0.7		>100
7	OEt	OH	H	H	OH	3.2 ± 0.7		>100
8	OMe	OH	H	OH	H	1.7 ± 0.25		>100
9	OMe	OH	OH	H	H	0.50 ± 0.04		>100

IC_50_ curves were generated from 3 technical replicates at each concentration of test compound and at least 2 biological replicates. Results depicted as mean ± SEM.

^a^Z-isomer.

**Table 3:** Effect of isoflavones on inhibition (IC_50_) of the epoxide hydrolase (LTB_4_ generation) and aminopeptidase (PGP degradation) activities of LTA_4_H as described in Materials and methods.

Cmpd. No.	R_1_	R_2_	R_3_	Epoxide Hydrolase IC_50_ ± SEM (μM)	Aminopeptidase IC_50_ ± SEM (μM)
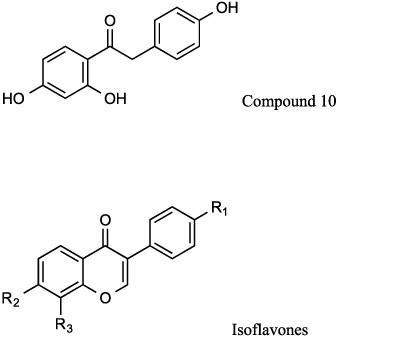					
10				34.24 ± 4.7	>100
11	OH	OH	H	15.0 ± 2.3	>100
12	OH	OH	OH	7.3 ± 0.8	62.7 ± 13.3
13	H	OH	H	48.2 ± 14.6	>100
14	OH	O-CH_2_-O		20.6 ± 2.4	20.6 ± 3.3
15	OH	Me	OH	1.2 ± 0.21	69.2 ± 10.9

IC_50_ curves were generated from 3 technical replicates at each concentration of test compound and at least 2 biological replicates. Results depicted as mean ± SEM.
