# Supplementary material for: The development of novel LTA4H modulators to selectively target LTB4 generation
Source: Sci Rep. 2017 Mar 17;7:44449. doi: 10.1038/srep44449 (PMC5355877; doi:10.1038/srep44449)
Supplement: Supplementary Information [file srep44449-s1.pdf]

**The development of novel $LTA_4H$ modulators to selectively target $LTB_4$ generation.**

Caroline M. Low[1], Samia Akthar[2], Dhiren F. Patel[2], Stephan Löser[2], Chi-Tung Wong[3], Patricia L. Jackson[4,5], J. Edwin Blalock[4], Stephen A. Hare[3], Clare M. Lloyd[2], Robert J. Snelgrove[2*].

**Supplementary Information**

**Supplementary Results**

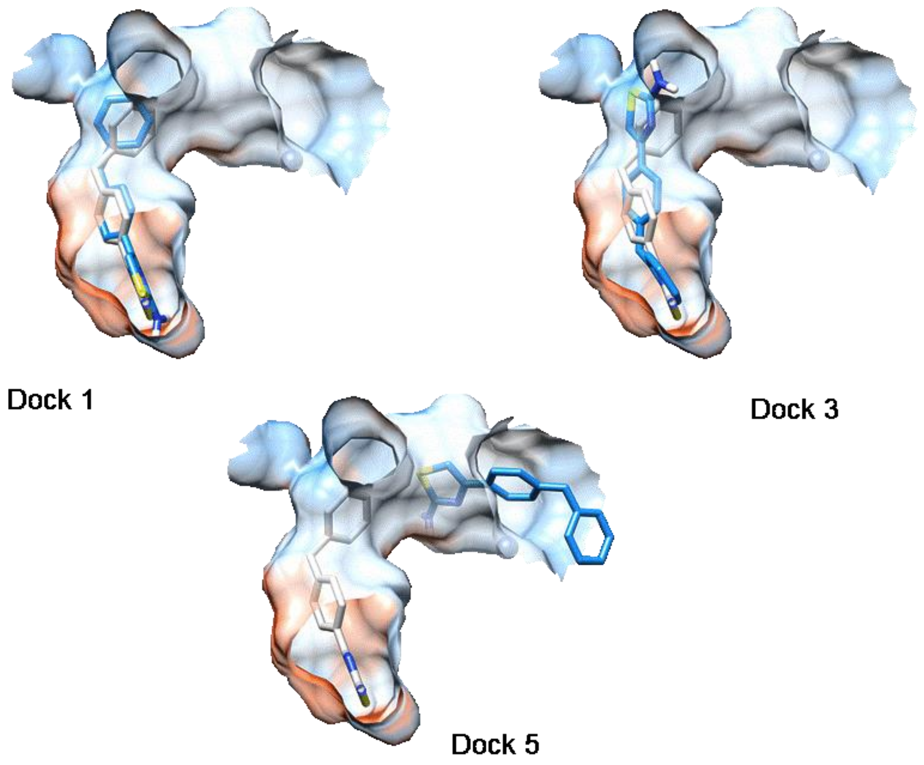


**Supplementary Figure S1. Potential of ARM1 to bind in peptide binding site of $LTA_4H$**

Examples of typical poses obtained for docking ARM1 into the active site of $LTA_4H$ with Autodock Vina under conditions described (see Methods for details). Binding of ARM1 revealed by X-ray crystallography (4MKT.pdb & 4L2L.pdb) is depicted in white, with ARM1 poses revealed by docking depicted in blue. A total of 5 poses were generated, numbered from the best scoring pose (dock 1) to lowest (dock 5). Poses not shown are closely related to those illustrated. Lower scoring poses clearly occupy the PGP binding site (dock 5), whereas higher scoring poses (dock 1) reflect the published X-ray data (4MKT.pdb & 4L2L.pdb). This dynamic behaviour may reflect our observations that ARM1 is a selective, but not completely specific inhibitor of the hydrolase activity of $LTA_4H$. The protein surface is coloured by amino acid hydrophobicity from most hydrophobic (orange) to hydrophilic (blue) through white regions.

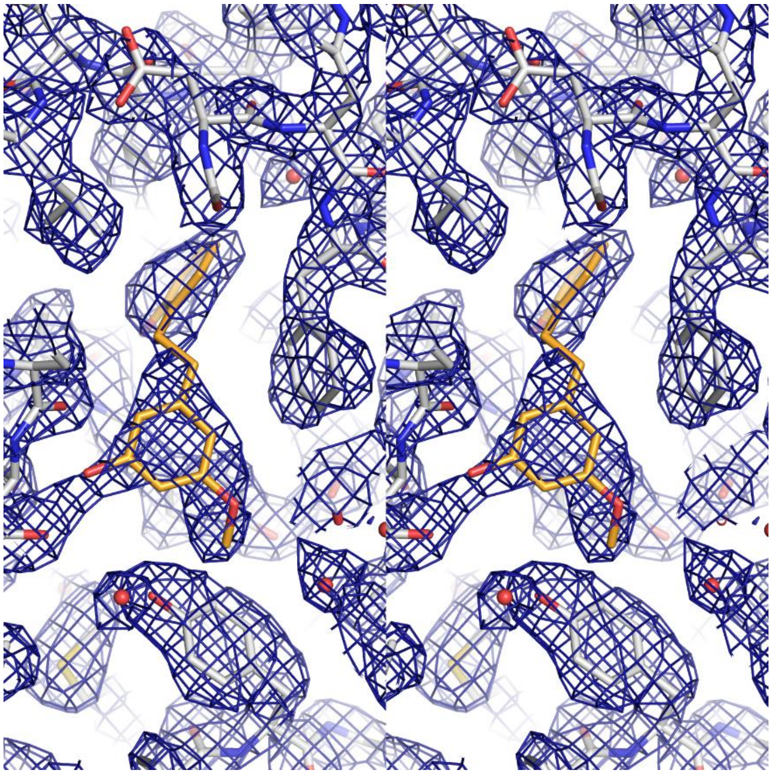

**Supplementary Figure S2.** Wall-eye stereo figure showing the electron density (2Fo-Fc map at 1σ shown as blue mesh) surrounding compound 9 (orange sticks). Nearby protein atoms are shown as light-grey sticks

| | **Compound 9** | **Compound 12** |
|---|---|---|
| **$logD_{pH7.4}$** | 2.25 ± 0.10 | 1.24±0.06 |
| **Mouse plasma protein binding** | 97.87% | 92.77% |
| **Human plasma protein binding** | 97.75% | 96.74% |
| **Mouse liver microsomes** | $Cl_{int}$ 465.9 ± 100.0 µL/min/mg protein<br>$t_{1/2}$ 1.5 min | $Cl_{int}$ 8.9 ± 0.7 µL/min/mg protein<br>$t_{1/2}$ 78.2 ± 6.1 min |
| **Human liver microsomes** | $Cl_{int}$ 59.5 µL/min/mg protein<br>$t_{1/2}$ 11.7 min | $Cl_{int}$ <5 µL/min/mg protein<br>$t_{1/2}$ >139 min |

**Supplementary Table S1**: *in vitro* ADMET profiles of lead series

**Supplementary Table S2: Data collection and refinement statistics**

| | Lta4h:Compound9 |
|---|---|
| **Data collection** | |
| Space group | $P2_12_12_1$ |
| Cell dimensions | |
| *a*, *b*, *c* (Å) | 78.25, 87.05, 99.96 |
| α, β, γ (°) | 90.0, 90.0, 90.0 |
| Resolution (Å) | 100 – 2.30 (2.42 – 2.30)* |
| $R_{merge}$ | 0.142 (0.497) |
| $I / \sigma I$ | 5.9 (1.9) |
| Completeness (%) | 96.4 (94.1) |
| Redundancy | 3.8 (3.3) |
| | |
| **Refinement** | |
| Resolution (Å) | 40 – 2.3 |
| No. reflections | 28042 |
| $R_{work}$ / $R_{free}$ | 0.240 / 0.288 |
| No. atoms | |
| Protein | 4844 |
| Ligand/ion | 29 |
| Water | 437 |
| B-factors | |
| Protein | 27.3 |
| Ligand/ion | 38.0 |
| Water | 27.7 |
| R.m.s. deviations | |
| Bond lengths (Å) | 0.013 |
| Bond angles (°) | 1.712 |

*Values in parentheses are for highest-resolution shell.

**Supplementary Experimental Procedures**

<u>Compound synthesis</u>

Compounds were custom synthesized by Peakdale Molecular as described below.

**Synthesis of compounds 6, 8 and 9**

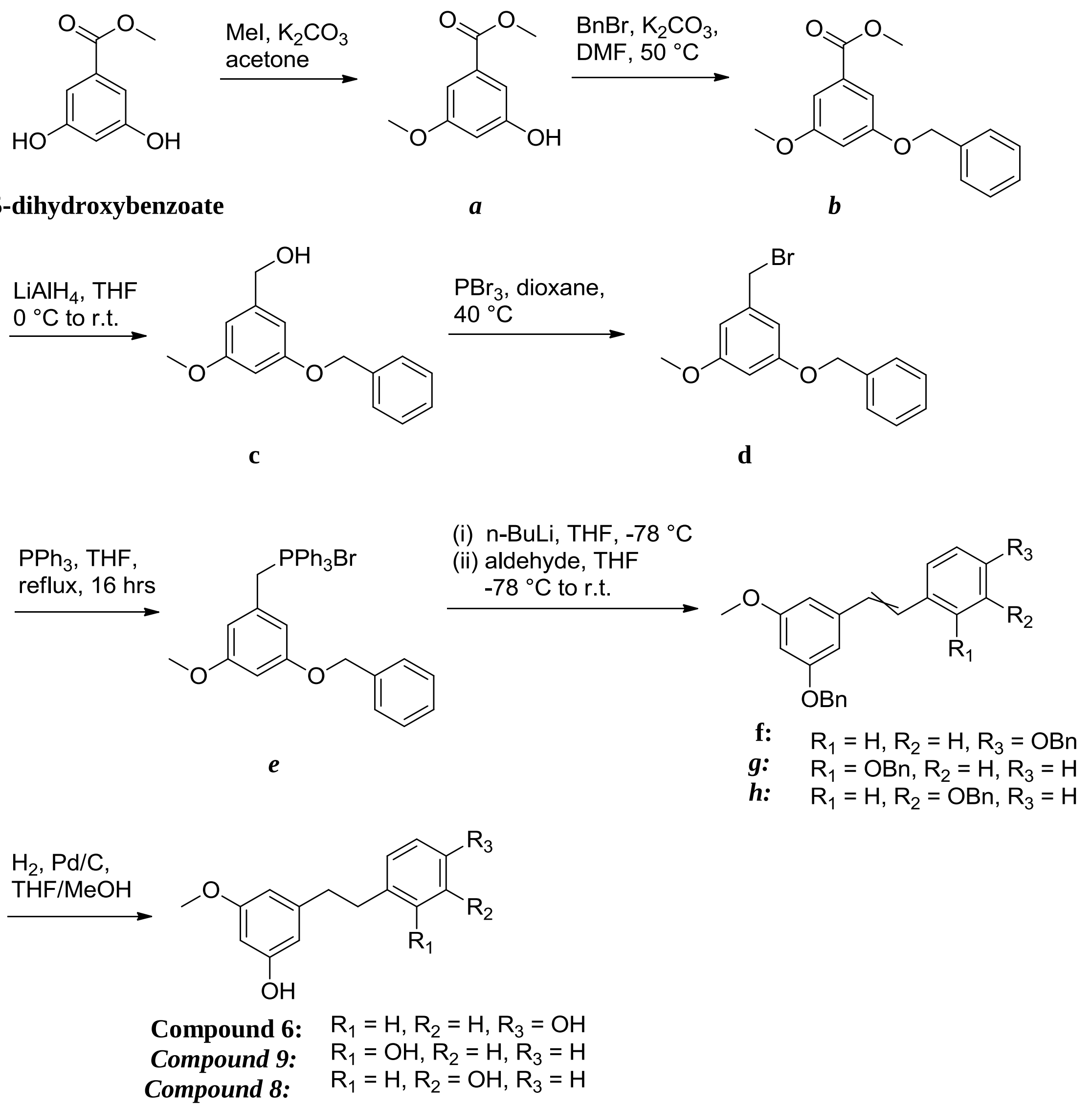


*Synthesis of methyl 3-hydroxy-5-methoxybenzoate (**a**)*

To a stirred suspension of methyl 3,5-dihydroxybenzoate (10 g, 59.5 mmol) and potassium carbonate (9.05 g, 65.45 mmol) in acetone (300 mL) was added iodomethane (3.7 mL, 59.5 mmol) at 0 °C. The mixture

was stirred at RT for 16 h, and then refluxed for a further 24 h. The mixture was then cooled to RT, and filtered through a pad of Celite. The filtrate was evaporated to give an orange oil, which was purified via flash chromatography (eluant: 9:1 to 8:2 gradient of heptane/ethyl acetate), affording ***a*** (3.5 g, 33% yield).

$^{1}$H NMR ($CDCl_3$, 300 MHz): δ 7.16 – 7.19 (m, 1H), 7.14 – 7.16 (m, 1H), 6.62 (t, 1H), 5.67 – 5.76 (br, 1H), 3.91 (s, 3H), 3.81 (s, 3H).

LCMS (basic, 1.2 min): RT = 0.57 min, [M+H]+ 183, purity 100%.

*Synthesis of methyl 3-(benzyloxy)-5-methoxybenzoate (**b**)*

To a stirred suspension of methyl 3-hydroxy-5-methoxybenzoate ***a*** (3.5 g, 19.2 mmol) and potassium carbonate (5.3 g, 38.4 mmol) in N,N-dimethylformamide (100 mL) was added benzyl bromide (2.3 mL, 19.2 mmol). The mixture was stirred at 60 °C for 18 h. The mixture was then diluted with ethyl acetate (250 mL) and water (250 mL), and the layers separated. The organic extract was washed with brine (3 x 200 mL), dried over sodium sulfate, and evaporated to give a brown oil, which was purified via flash chromatography (eluant: 19:1 to 37:3 gradient of heptane/ethyl acetate), affording ***b*** (3.8 g, 73% yield).

$^{1}$H NMR ($CDCl_3$, 400 MHz): δ 7.30 – 7.45 (m, 5H), 7.27 (dd, 1H, J = 2.3 Hz & 1.4 Hz), 7.19 (dd, 1H, J = 2.3 Hz & 1.4 Hz), 6.71 (t, 1H, J = 2.3 Hz), 5.07 (s, 2H), 3.90 (s, 3H), 3.81 (s, 1H).

LCMS (basic, 1.2 min): RT = 0.91 min, [M+H]+ 273, purity 99.2%.

*Synthesis of (3-(benzyloxy)-5-methoxyphenyl)methanol (**c**)*

To a suspension of lithium aluminium hydride (534 mg, 14.08 mmol) in THF (20 mL) was added ***b*** (3.8 g, 14.08 mmol) in THF (60 mL), dropwise, with ice/salt bath cooling to ensure internal temperature did not rise above 10 °C during addition. After addition was complete, the mixture was stirred at RT for 2 h. Excess lithium aluminium hydride was then quenched by careful addition of sodium sulfate decahydrate. After stirring the suspension for 15 min, ethyl acetate (150 mL) was added, and the suspension filtered through Celite. The filtrate was evaporated to give ***c*** (3.38 g, 98% yield) as a colourless oil.

$^{1}H$ NMR ($CDCl_3$, 400 MHz): δ 7.30 – 7.45 (m, 5H), 6.60 (s, 1H), 6.54 (s, 1H), 6.46 (t, 1H, J = 2.3 Hz), 5.04 (s, 2H), 4.63 (s, 2H), 3.78 (s, 3H)

LCMS (basic, 1.2 min): RT = 0.76 min, [M+H]+ 245, purity 95.3%.

*Synthesis of 1-(benzyloxy)-3-(bromomethyl)-5-methoxybenzene (**d**)*

A solution of ***c*** (3.38 g, 13.85 mmol) and phosphorus tribromide (1.5 mL, 15.23 mmol) in dioxane (35 mL) was heated at 40 °C for 2 h. After cooling to RT, the mixture was poured into saturated aqueous sodium bicarbonate solution (100 mL), which was then extracted with ethyl acetate (2 x 100 mL). The combined organic extracts were dried over sodium sulfate and evaporated to give ***d*** (3.89 g, 92% yield).

$^{1}H$ NMR ($CDCl_3$, 400 MHz): δ 7.30 – 7.45 (m, 5H), 6.62 (s, 1H), 6.54 (s, 1H), 6.46 (t, 1H, J = 2.3 Hz), 5.03 (s, 2H), 4.41 (s, 2H), 3.77 (s, 3H).

LCMS (basic, 1.2 min): RT = 0.95 mins, [M+H]+ 307/309, purity 94%.

*Synthesis of (3-(benzyloxy)-5-methoxybenzyl)triphenylphosphonium bromide (**e**)*

A solution of ***d*** (3.89 g, 12.67 mmol) and triphenylphosphine (3.32 g, 12.67 mmol) in THF (80 mL) was stirred at 70 °C for 18 h. The reaction mixture was then cooled to RT, and the resulting white crystals collected via Buchner filtration, washing the filter cake with THF (2 x 15 mL) to afford the phosphonium bromide salt ***e*** (4.95 g, 69% yield).

$^{1}H$ NMR (DMSO-$d^6$, 300 MHz): δ 7.85 – 7.94 (m, 3H), 7.60 – 7.76 (m, 12H), 7.25 – 7.40 (m, 5H), 6.48 (s, 1H), 6.19 (s, 1H), 6.09 (s, 1H), 5.04 (d, 2H, J = 15.5 Hz), 4.79 (s, 2H), 3.45 (s, 3H).

$^{31}P$ NMR (DMSO-d6, 121 MHz): δ 23.82 (s, 1P)

LCMS (basic, 1.2 min): RT = 1.00 min, [M+H]+ 489 (phosphorane), purity 97.4%.

*Synthesis of 1-(benzyloxy)-3-(4-(benzyloxy)styryl)-5-methoxybenzene (**f**)*

A solution of ***e*** (250 mg, 0.438 mmol) in THF (5 mL) was cooled to -78 °C, and n butyl lithium (2M in hexanes, 0.23 mL, 0.460 mmol) was added dropwise, maintaining internal temperature below -65 °C. This

afforded a bright yellow suspension. After stirring for 2 h at -78 °C, a solution of 4-(benzyloxy)benzaldehyde (92 mg, 0.438 mmol) in THF (5 mL) was added dropwise. After addition was complete, the yellow suspension was allowed to warm slowly to RT, during which time the bright yellow suspension became a pale yellow solution. 2h after the addition, the mixture was diluted with water (30 mL), and extracted with dichloromethane (3 x 25 mL); the combined organic extracts were dried over sodium sulfate and evaporated to give a yellow oil, which was purified by flash chromatography (eluant: 19:1 heptane/ethyl acetate) to give ***f*** (148 mg, 80% yield).

$^1$H NMR ($CDCl_3$, 400 MHz): δ 7.30 – 7.47 (m, 10H), 6.40 – 7.05 (m, 9H, overlapping 5:3 mixture of E and Z isomers), 5.08, 5.07, 5.03, 4.89 (4 x s, 4H), 3.81, 3.64 (2 x s, 3H).

LCMS (basic, 1.2 min): RT = 1.09 and 1.10 min, [M+H]+ 423, purity 42.6% and 53.5%, total 96.1%.

*Synthesis of 3-(4-hydroxyphenethyl)-5-methoxyphenol (**compound 6**)*

To a solution of ***f*** (148 mg, 0.351 mmol) in THF/methanol (1:1 ratio (v/v), 5 mL) in an autoclave was added 5% palladium on charcoal (10 mg), which was then pressurised to 400 psi with hydrogen. After 3h, the reaction was complete, so the catalyst was then removed via filtration through Celite, and the filtrate evaporated to give a dark green oil, which was purified by flash chromatography (eluant: 19:1 dichloromethane/methanol) to afford ***compound 6*** (60 mg, 70% yield).

$^1$H NMR ($CDCl_3$, 300 MHz): 7.03 (d, 2H, J = 8.7 Hz), 6.74 (d, 2H, J = 8.7 Hz), 6.32 (s, 1H), 6.24 (d, 2H, J = 1.4 Hz), 4.70 (s, 1H), 4.63 (s, 1H), 3.75 (s, 3H), 2.75 – 2.85 (m, 4H).

LCMS (basic, 4.7 min, 0-50% gradient): RT = 2.46 min, [M+H]+ 245, purity 98.9%.

*Synthesis of 1-(benzyloxy)-3-(2-(benzyloxy)styryl)-5-methoxybenzene (**g**)*

A solution of ***e*** (500 mg, 0.876 mmol) in THF (10 mL) was cooled to -78 °C, and *n*-butyl lithium (2M in hexanes, 0.46 mL, 0.92 mmol) was added dropwise, maintaining internal temperature below -65 °C. This afforded a bright yellow suspension. After stirring for 2 h at -78 °C, a solution of 2-(benzyloxy)benzaldehyde (184 mg, 0.876 mmol) in THF (10 mL) was added dropwise. After addition was

complete, the yellow suspension was allowed to warm slowly to RT, during which time the bright yellow suspension became a pale yellow solution. Two h after the addition, the mixture was diluted with water (30 mL), and extracted with dichloromethane (3 x 25 mL); the combined organic extracts were dried over sodium sulfate and evaporated to give a yellow oil, which was purified by flash chromatography (eluant: 19:1 heptane/ethyl acetate) to give ***g*** (230 mg, 62% yield).

$^{1}$H NMR ($CDCl_3$, 400 MHz): 7.28 – 7.61 (m, 10H), 6.34 – 7.25 (m, 9H, overlapping mixture of E and Z isomers), 5.15, 5.08, 5.05, 4.79 (4 x s, 4H), 3.80, 3.59 (2 x s, 3H).

LCMS (basic, 1.2 min): RT = 1.10 and 1.11 min, [M+H]+ 423, purity 55.1% and 38.2%, total 93.3%.

*Synthesis of 3-(2-hydroxyphenethyl)-5-methoxyphenol (**compound 9**)*

To a solution of ***g*** (230 mg, 0.545 mmol) in THF/methanol (1:1 ratio (v/v), 10 mL) in an autoclave was added 5% palladium on charcoal (10 mg), which was then pressurized to 500 psi with hydrogen, and stirred at RT for 30 h. The catalyst was then removed via filtration through Celite, and the filtrate evaporated to give a dark green oil, which was purified by flash chromatography (eluant: 96:4 dichloromethane/methanol) to afford ***compound 9*** (91 mg, 68% yield).

$^{1}$H NMR ($CDCl_3$, 400 MHz): δ 7.06 – 7.11 (m, 2H), 6.86 (t, 1H, J = 6.5 Hz), 6.74 (d, 1H, J = 9 Hz), 6.33 (s, 1H), 6.27 (s, 1H), 6.24 – 6.27 (m 1H), 4.69 (s, 1H), 4.59 (s, 1H), 3.74 (s, 3H), 2.78 – 2.92 (m, 2H).

LCMS (basic, 11 min): RT = 5.77 min, [M+H]+ 245, purity 97.2%.

*Synthesis of 1-(benzyloxy)-3-(3-(benzyloxy)styryl)-5-methoxybenzene (**h**)*

A solution of ***e*** (500 mg, 0.876 mmol) in THF (10 mL) was cooled to -78 °C, and *n*-butyl lithium (2M in hexanes, 0.46 mL, 0.92 mmol) was added dropwise, maintaining internal temperature below -65 °C. This afforded a bright yellow suspension. After stirring for 2 h at -78 °C, a solution of 3-(benzyloxy)benzaldehyde (184 mg, 0.876 mmol) in THF (10 mL) was added dropwise. After addition was complete, the yellow suspension was allowed to warm slowly to RT, during which time the bright yellow suspension became a pale yellow solution. Two hours after the addition, the mixture was diluted with water

(30 mL), and extracted with dichloromethane (3 x 25 mL); the combined organic extracts were dried over sodium sulfate and evaporated to give a yellow oil, which was purified by flash chromatography (eluant: 19:1 heptane/ethyl acetate) to give ***h*** (190 mg, 51% yield).

$^{1}$H NMR ($CDCl_3$, 400 MHz): δ 7.25 – 7.50 (m, 10H), 6.37 – 7.18 (m, 9H), 5.10, 5.08, 4.91, 4.86 (4 x s, 4H), 3.82, 3.63 (2 x s, 3H).

LCMS (basic, 1.2 min): RT = 1.10 min, [M+H]+ 423, purity 94.1%.

*Synthesis of 3-(3-hydroxyphenethyl)-5-methoxyphenol (**compound 8**)*

To a solution of ***h*** (190 mg, 0.45 mmol) in THF/methanol (1:1 ratio (v/v), 10 mL) in an autoclave was added 5% Palladium on charcoal (10 mg), which was then pressurized to 500 psi with hydrogen, and stirred at RT for 48 h. The catalyst was then removed via filtration through Celite, and the filtrate evaporated to give a brown oil, which was purified by flash chromatography (96:4 dichloromethane/methanol) to afford ***compound 8*** (55 mg, 50% yield).

$^{1}$H NMR ($CDCl_3$, 400 MHz): 7.14 (t, 1H, J = 9.5Hz), 6.75 (d, 1H, J = 7.8 Hz), 6.63 – 6.68 (m, 2H), 6.31 (s, 1H), 6.22 – 6.27 (m, 2H), 4.61 – 4.70 (m, 2H, phenolic OHs), 3.74 (s, 3H), 2.77 – 2.87 (m, 4H).

LCMS (basic, 4.7 min): RT = 1.88 min, [M+H]+ 245, purity 100%.

**Synthesis of compound 7**

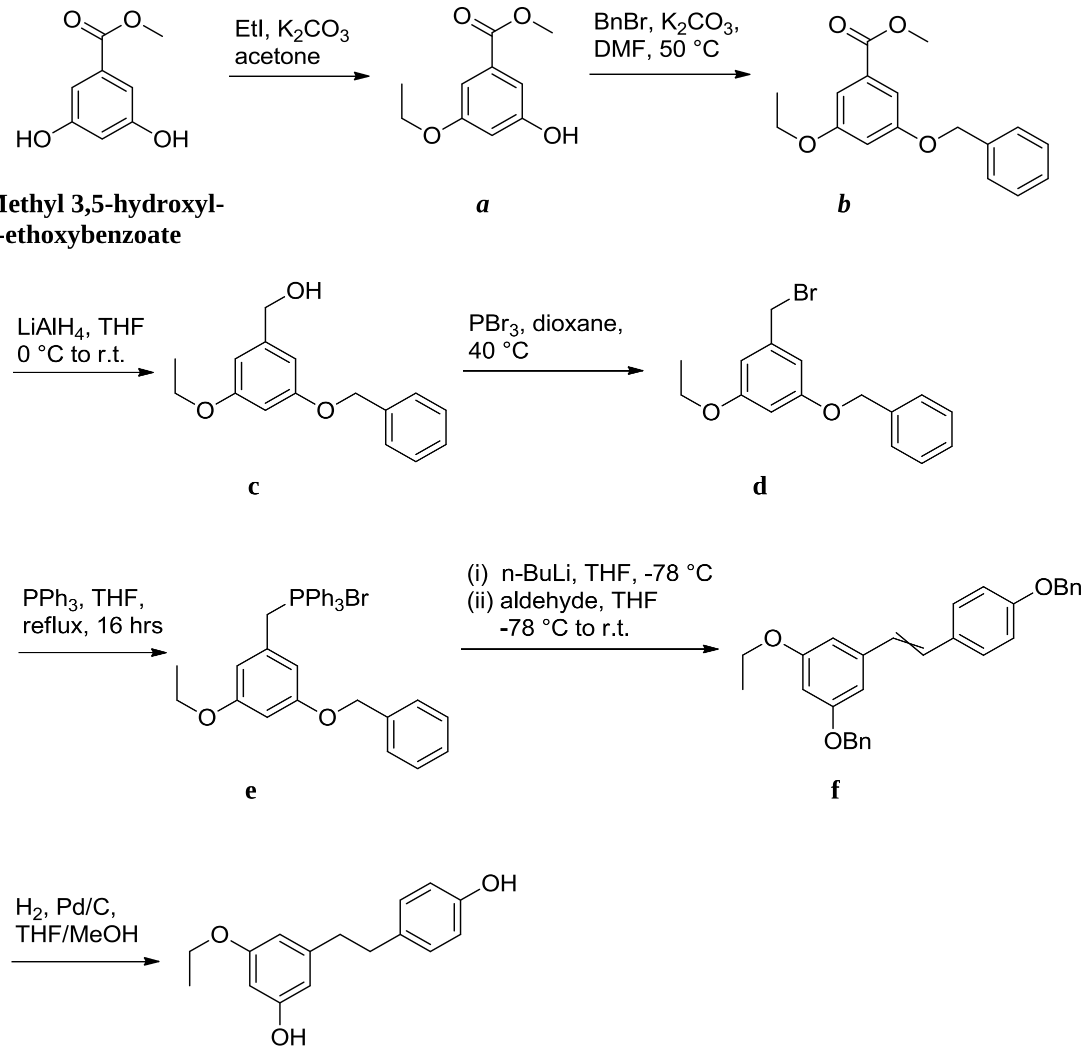


**Compound 7**

*Synthesis of methyl 3-hydroxy-5-ethoxybenzoate (**a**)*

To a stirred suspension of methyl 3,5-dihydroxybenzoate (4 g, 23.8 mmol) and potassium carbonate (9.05 g, 26.1 mmol) in acetone (130 mL) was added iodoethane (1.9 mL, 23.8 mmol) at 0 °C. The mixture was stirred at RT for 18 h, and then refluxed for a further 24 h. The mixture was then cooled to RT, and filtered

through a pad of Celite. The filtrate was evaporated to give an orange oil, which was purified via flash chromatography (eluant: 9:1 to 8:2 gradient of heptane/ethyl acetate), affording ***a*** (1.38 g, 30% yield).

$^1$H NMR ($CDCl_3$ 300 MHz): δ 7.11-7.16 (m 2H), 6.60 (t, 1H, J = 2.3 Hz), 5.27 (s, 1H), 4.04 (q, 2H, J = 6.9 Hz), 1.41 (t, 3H, J = 6.9 Hz).

LCMS (basic, 1.2 min): RT = 0.64 min, [M+H]+ 197, purity 100%.

*Synthesis of methyl 3-(benzyloxy)-5-ethoxybenzoate (**b**)*

To a stirred suspension of methyl 3-hydroxy-5-ethoxybenzoate ***a*** (1.38 g, 7.04 mmol) and potassium carbonate (1.94 g, 14.08 mmol) in N,N-dimethylformamide (50 mL) was added benzyl bromide (2.3 mL, 7.04 mmol). The mixture was stirred at 60 °C for 18 h. The mixture was then diluted with ethyl acetate (250 mL) and water (250 mL), and the layers separated. The organic extract was washed with brine (3 x 200 mL), dried over sodium sulfate, and evaporated to give a brown oil, which was purified via flash chromatography (eluant: 19:1 to 37:3 gradient of heptane/ethyl acetate), affording ***b*** (1.11 g, 55% yield).

$^1$H NMR ($CDCl_3$ 300 MHz): δ 7.30 – 7.45 (m, 5H), 7.25 – 7.28 (m, 1H), 7.19 (dd, 1H, J = 2.3 Hz and 1.4 Hz), 6.71 (t, 1H, J = 2.3 Hz), 5.07 (s, 2H), 4.04 (q, 2H, J = 7.3 Hz), 3.90 (s, 3H), 1.41 (t, 3H, J = 7 Hz).

LCMS (basic, 1.2 mins): RT = 0.96 min, [M+H]+ 287, purity 98.3%.

*Synthesis of (3-(benzyloxy)-5-ethoxyphenyl)methanol (**c**)*

To a suspension of lithium aluminium hydride (147 mg, 3.88 mmol) in THF (10 mL) was added ***b*** (1.11 g, 3.88 mmol) in THF (15 mL), dropwise, with ice/salt bath cooling to ensure internal temperature did not rise above 10°C during addition. After addition was complete, the mixture was stirred at RT for 2 h. Excess lithium aluminium hydride was then quenched by careful addition of sodium sulfate decahydrate. After stirring the suspension for 15 min, ethyl acetate (50 mL) was added, and the suspension filtered through Celite. The filtrate was evaporated to give ***c*** (968 mg, 97% yield) as a colourless oil.

$^{1}H$ NMR ($CDCl_3$, 400 MHz): δ 7.30 – 7.45 (m, 5H), 6.59 (s, 1H), 6.52 (s, 1H), 6.45 (t, 1H, J = 2.3 Hz), 5.03 (s, 2H), 4.62 (s, 2H), 4.00 (q, 2H, J = 6.9 Hz), 1.39 (t, 3H, J = 6.9 Hz).

LCMS (basic, 1.2 min): RT = 0.89 min, [M+H]+ 259, purity 88.9%.

*Synthesis of 1-(benzyloxy)-3-(bromomethyl)-5-ethoxybenzene (**d**)*

A solution of ***c*** (968 mg, 3.75 mmol) and phosphorus tribromide (0.38 mL, 4.12 mmol) in dioxane (10 mL) was heated at 40 °C for 2 h. After cooling to RT, the mixture was poured into saturated aqueous sodium bicarbonate solution (100mL), which was then extracted with ethyl acetate (2 x 100 mL). The combined organic extracts were dried over sodium sulfate and evaporated to give ***d*** (1.18 g, 98% yield).

$^{1}H$ NMR ($CDCl_3$, 400 MHz): δ 7.32 – 7.45 (m, 5H), 6.60 (s, 1H), 6.54 (s, 1H), 6.46 (t, 1H, J = 2.3 Hz), 5.02 (s, 2H), 4.40 (s, 2H), 4.00 (q, 2H, J = 6.9 Hz), 1.39 (t, 3H, J = 7.3 Hz).

LCMS (basic, 1.2 min): RT = 0.99 mins, [M+H]+ 321/323, purity 95.1%.

*Synthesis of (3-(benzyloxy)-5-ethoxybenzyl)triphenylphosphonium bromide (**e**)*

A solution of ***d*** (1.18 g, 3.67 mmol) and triphenylphosphine (960 mg, 3.67 mmol) in THF (30 mL) was stirred at 70 °C for 18 h. The reaction mixture was then cooled to RT, and the resulting white crystals collected via Buchner filtration, washing the filter cake with THF (2 x 8 mL) to afford the phosphonium bromide salt ***e*** (865 mg, 41% yield).

$^{1}H$ NMR (DMSO-$d^6$, 300 MHz): δ 7.85 – 7.94 (m, 3H), 7.60 – 7.77 (m, 12H), 7.23 – 7.39 (m, 5H), 6.46 (s, 1H), 6.16 (s, 1H), 6.11 (s, 1H), 5.04 (d, 2H, J = 15.5 Hz), 3.67 (q, 2H, J = 7.3 Hz), 1.13 (t, 3H, J = 7 Hz).

$^{31}P$ NMR (DMSO-$d^6$, 121 MHz): δ 23.82 (s, 1P).

LCMS (basic, 1.2 min): RT = 1.02 min, [M+H]+ 503 (phosphorane), purity 91%.

*Synthesis of 1-(benzyloxy)-3-(4-(benzyloxy)styryl)-5-ethoxybenzene (**f**)*

A solution of ***e*** (500 mg, 0.856 mmol) in THF (10 mL) was cooled to -78 °C, and n-butyl lithium (2M in hexanes, 0.45 mL, 0.90 mmol) was added dropwise, maintaining internal temperature below -65 °C. This afforded a bright yellow suspension. After stirring for 2 h at -78 °C, a solution of 4-(benzyloxy)benzaldehyde (181 mg, 0.856 mmol) in THF (10 mL) was added dropwise. After addition was complete, the yellow suspension was allowed to warm slowly to RT, during which time the bright yellow suspension became a pale yellow solution. Two h after the addition, the mixture was diluted with water (50 mL), and extracted with dichloromethane (3 x 40 mL); the combined organic extracts were dried over sodium sulfate and evaporated to give a yellow oil, which was purified by flash chromatography (eluant: 19:1 heptane/ethyl acetate) to give ***f*** (222 mg, 80% yield).

$^1$H NMR ($CDCl_3$, 400 MHz): δ 7.28 – 7.48 (m, 10H), 7.21 (d, 1H, J = 8 Hz), 6.38 – 7.05 (m, 8H), 5.09, 5.07, 5.03, 4.88 (4 x s, 4H), 4.03, 3.86 (2 x q, 2H, J = 6.9 Hz), 1.41, 1.32 (2 x t, 3H, J = 8 Hz).

LCMS (basic, 1.2 min): RT = 1.12 and 1.13 min, [M+H]+ 437, purity 42.3% and 55.6%, total purity 97.9%.

*Synthesis of 1-(benzyloxy)-3-(4-(benzyloxy)styryl)-5-ethoxybenzene (**compound 7**)*

A solution of ***f*** (222 mg, 0.509 mmol) in THF/methanol (1:1 ratio (v/v), 6 mL) was charged into a 20 mL microwave vial, equipped with a stirrer bar. 5% palladium on charcoal (10 mg) was added, and the vial capped. The septum was pierced, and a flat-ended needle pushed through the piercing. The vial was transferred to an autoclave, which was pressurized to 500 psi with hydrogen, and stirred at RT for 72 h. The catalyst was then removed via filtration through Celite, and the filtrate evaporated to give a yellow oil, which was purified by flash chromatography (eluant: 24:1 dichloromethane/methanol) to afford a pale yellow oil, which was further purified by mass-directed prep HPLC (Waters XBridge C18 19x100mm column, 5μm particle size, gradient from 70:30 to 50:50 of 10mM NH4HCO3 (aq)/acetonitrile, 20 mL/min) to give ***compound 7*** (69 mg, 52% yield).

$^1$H NMR ($CDCl_3$, 400 MHz): δ 7.03 (d, 2H, J = 8.7 Hz), 6.73 (d, 2H, J = 8.3 Hz), 6.31 (s, 1H), 6.20 – 6.24 (m, 2H), 4.57 (s, 1H), 4.53 (s, 1H), 3.96 (q, 2H, J = 6.8 Hz), 2.73 – 2.85 (m, 4H), 1.38 (t, 3H, J = 6.8 Hz).

LCMS (basic, 4.7 min): RT = 2.00 min, [M+H]+ 259, purity 96.8%.

**Synthesis of compound 14**

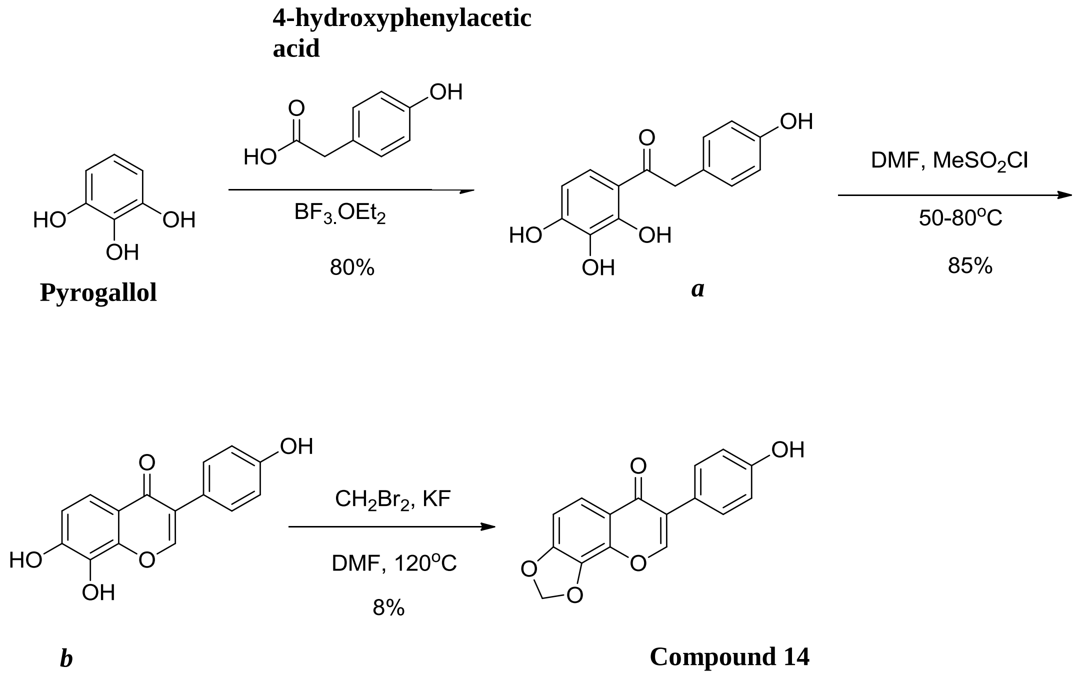


*Synthesis of 2-(4-hydroxyphenyl)-1-(2,3,4-trihydroxyphenyl)ethanone (**a**)*

A mixture of pyrogallol (2.0 g, 15.86 mmol) and 4-hydroxyphenylacetic acid (2.4 g, 15.9 mmol) in boron trifluoride diethyl etherate (16 ml) was heated at 85 °C for 3.5h. The reaction mixture was cooled and poured into ice cold NaOAc (10% aqueous; 500 ml). The mixture was stirred overnight and the product was then collected by filtration, washed with water (3 x 50 ml) and dried in a vacuum at 50 °C over KOH to give ***a*** as a brown solid (3.32 g, 80%).

UPLC (basic, 1.20 min): RT 0.43 min, [$M-H^+$] 259, purity 68%.

A sample was purified by passing through a silica pad (eluent: methyl-THF) to give ***a*** as a light brown solid.

UPLC (basic, 1.20 min): RT 0.40 min, [$M-H^+$] 259, purity 87%.

*Synthesis of 7,8-dihydroxy-3-(4-hydroxyphenyl)-4H-chromen-4-one (**b**)*

***a*** (1.0 g, 3.84 mmol) was dissolved in DMF (8 ml) and stirred at 50 °C under argon for 10 min. Methanesulfonyl chloride (0.9 ml; 11.5 mmol) was then added and the reaction mixture heated at 80 °C for

1.5 h. The reaction mixture was cooled in an ice/MeOH/salt bath, quenched with water (65 ml) and extracted into diethyl ether (160 ml). The aqueous phase was extracted again with diethyl ether (2 x 160 ml) and the combined extracts washed with brine (2 x 80 ml), $NaHCO_3$ (sat.) (80 ml), dried ($Na_2SO_4$) and concentrated to give ***b*** as a light brown solid (880 mg, 85%).

UPLC (basic; 0-50% MeCN; 1.20 min): RT 0.37 min $[M+H]^+$ 271, purity 55%.

*Synthesis of 7-(4-hydroxyphenyl)-6H-[1,3]dioxolo[4,5-h]chromen-6-one (**compound 14**)*

Potassium fluoride (206 mg, 3.55 mmol), followed by dibromomethane (358 µl, 5.11 mmol) were added to a stirred solution of ***b*** (600 mg, 2.22 mmol) in DMF (27 ml) and heated at 120 °C under argon for 3 h. The reaction mixture was cooled to RT and partitioned between water (60 ml) and EtOAc (250 ml). The organic phase was then washed with water (3 x 60 ml), brine (60 ml), dried ($Na_2SO_4$) and concentrated to a brown solid (550 mg). The crude product was suspended in EtOAc (50 ml) and the insoluble material was removed by filtration. The filtrate was concentrated to a light brown solid (370 mg) and purified by mass directed auto purification HPLC (20 to 45% MeCN with 0.1% NH4OH over 10 min., RT: 5.2 – 8.2 min.) to give ***compound 14*** (50 mg, 8%) as a pale brown solid.

$^1$H NMR (300MHz., DMSO-$d_6$), 9.56 (brs , 1H), 8.35 (s, 1H), 7.66 (d, 1H), 7.36-7.33 (m , 2H), 7.14 (d, 1H), 6.83-6.77 (m, 2H), 6.28 (s, 1H).

UPLC (basic; 4.50 min): RT 1.76 min $[M+H]^+$ 283, purity 98%.

M.P. 279-281 °C

## Synthesis of compound 15

**4-hydroxyphenylacetic acid**

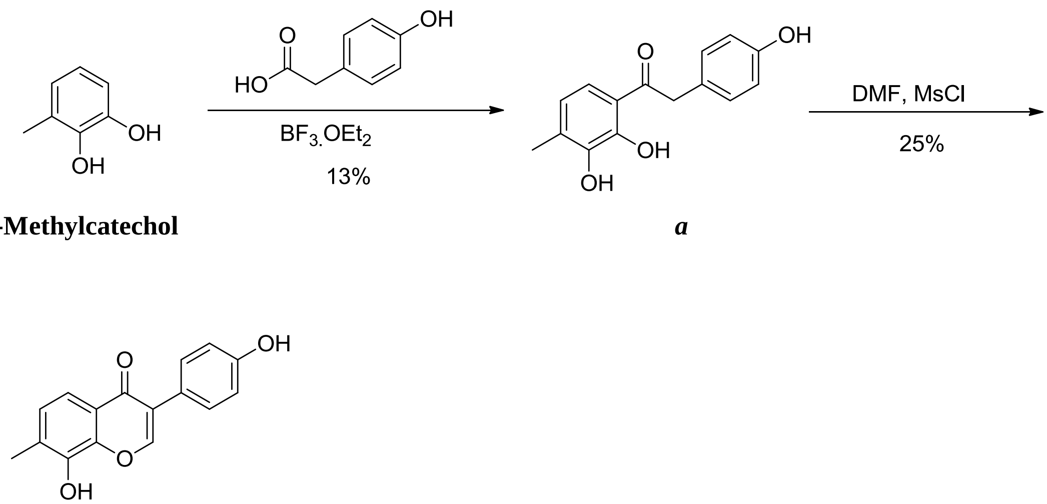


**3-Methylcatechol** ***a***

**Compound 15**

*Synthesis of 1-(2,3-dihydroxy-4-methylphenyl)-2-(4 hydroxyphenyl)ethanone (**a**)*

3-Methylcatechol (1 g, 8.06 mmol) and 4-hydroxyphenylacetic acid **(**1.16 g, 7.65 mmol) in boron trifluoride diethyl etherate (2.2 ml) were heated at 120°C for 10 min in a Biotage Initiator 60 microwave. The reaction was quenched with water (43 ml) and extracted into diethyl ether (3 x 50 ml). The combined organics were washed with brine (3 x 100 ml), $NaHCO_3$ (saturated aqueous; 3 x 100ml), dried ($Na_2SO_4$) and concentrated to a brown oil. The crude product was purified by flash chromatography (eluent: 20-35% EtOAc in heptane to give ***a*** as an orange oil (270 mg, 13%).

$^1$H NMR (300MHz., DMSO-d$^6$), 7.46-7.43 (m, 1H), 7.15-6.98 (m, 2H), 6.70-6.60 (m, 3H), 4.20 (s, 2H), 2.17 (s, 3H). UPLC (basic, 1.20 min): RT = 0.68 min, $[M+H]^+$259, purity 87%.

*Synthesis of 8-hydroxy-3-(4-hydroxyphenyl)-7-methyl-4H-chromen-4-one (**compound 15**)*

***a*** (57 mg, 0.22 mmol) was dissolved in DMF (0.5 ml) and stirred at 50 °C under argon for 10 min. Methanesulfonyl chloride (116 µl; 1.50 mmol) was then added in one portion and the reaction mixture

heated at 80 °C for 40 min. The reaction was cooled to RT, quenched with water (4 ml) and extracted into diethyl ether (10 ml). The aqueous phase was extracted again with diethyl ether (2 x 10 ml) and the combined extracts washed with brine (2 x 5 ml), $NaHCO_3$ (saturated aqueous; 2 x 5 ml), dried ($Na_2SO_4$) and concentrated to a light brown solid. The reaction was repeated to a total of 90 mg of crude material, which was then purified by mass directed auto purification HPLC (2 to 10% MeCN with 0.1% NH4OH over 10 min., RT: 3.5 – 6.2 min.) to give **compound 15** as an orange solid (30 mg, 25%).

$^1$H NMR (300MHz., DMSO-d$^6$), 9.54 (brs , 1H), 8.38 (s, 1H), 7.50-7.37 (m, 3H), 7.21 (d , 1H), 6.78 (d, 2H), 2.29 (s, 3H).UPLC (basic; 4.50 min): RT = 0.84 min [M+H]+ 269, purity 100%. M.P. 282-284 °C
